# Histone Modifications in a Mouse Model of Early Adversities and Panic Disorder: Role for *Asic1* and Neurodevelopmental Genes

**DOI:** 10.1038/srep25131

**Published:** 2016-04-28

**Authors:** Davide Cittaro, Valentina Lampis, Alessandra Luchetti, Roberto Coccurello, Alessandro Guffanti, Armando Felsani, Anna Moles, Elia Stupka, Francesca R. D’ Amato, Marco Battaglia

**Affiliations:** 1 Centre for Translational Genomics and Bioinformatics, San Raffaele Scientific Institute, Milan, Italy; 2 Developmental Psychopathology Unit, Vita-Salute San Raffaele University, Milan, Italy; 3 Institute of Cell Biology and Neurobiology, National Research Council/Fondazione Santa Lucia, Rome, Italy; 4 Laboratory of Molecular Neuroscience, Department of Biological Chemistry, The Edmond and Lily Safra Center of Brain Science, The Hebrew University of Jerusalem, Jerusalem, Israel; 5 Genomnia srl, Lainate, Italy; 6 Department of Psychiatry, University Of Toronto, Toronto, Canada; 7 Division of Child and Youth Mental Health, Centre for Addiction and Mental Health, Toronto, Canada

## Abstract

Hyperventilation following transient, CO_2_-induced acidosis is ubiquitous in mammals and heritable. In humans, respiratory and emotional hypersensitivity to CO_2_ marks separation anxiety and panic disorders, and is enhanced by early-life adversities. Mice exposed to the repeated cross-fostering paradigm (RCF) of interference with maternal environment show heightened separation anxiety and hyperventilation to 6% CO_2_-enriched air. Gene-environment interactions affect CO_2_ hypersensitivity in both humans and mice. We therefore hypothesised that epigenetic modifications and increased expression of genes involved in pH-detection could explain these relationships. Medullae oblongata of RCF- and normally-reared female outbred mice were assessed by ChIP-seq for H3Ac, H3K4me3, H3K27me3 histone modifications, and by SAGE for differential gene expression. Integration of multiple experiments by network analysis revealed an active component of 148 genes pointing to the *mTOR* signalling pathway and nociception. Among these genes, *Asic1* showed heightened mRNA expression, coherent with RCF-mice’s respiratory hypersensitivity to CO_2_ and altered nociception. Functional enrichment and mRNA transcript analyses yielded a consistent picture of enhancement for several genes affecting chemoception, neurodevelopment, and emotionality. Particularly, results with Asic1 support recent human findings with panic and CO_2_ responses, and provide new perspectives on how early adversities and genes interplay to affect key components of panic and related disorders.

In all mammals, an excess of CO_2_ in inhaled air induces hypercarbia and lowers blood pH: this relative acidosis stimulates ventilation, followed by enhanced arousal, and subsequent anxiety^1^,^2^. The mechanisms that underlie these responses include the activation of acid-sensing chemoreceptors, which have been identified in the brainstem^3^, perhaps most crucially in the ventral surface of the medulla oblongata Retrotrapezoid Nucleus^1^, and more recently in the amygdala^4^, with increased ventilation favoring the expulsion of excess CO_2_ and the reinstatement of systemic pH.Individuals differ from each other for the intensity of the physiological respiratory and anxious responses to heightened CO_2_ concentrations partially due to genetic factors, as shown by studies carried out in both animals and humans^5^,^6^. While the molecular genetic bases of CO_2_ sensitivity in man are largely unexplored, available quantitative genetics data are sufficient to establish connections between human responses to CO_2_ challenges and some behavioural phenotypes, and to support comparative experiments in animals. Children with Separation Anxiety Disorder (SAD) and adults with Panic Disorder (PD) -two genetically and developmentally-related anxiety disorders^7^ -, show responses to heightened CO_2_ concentrations that lie at the extreme of the distribution, having the paroxysmal features of panic-like anxiety and hyperventilation^8^. These exaggerated and specific responses can be documented early in life^7^, map the psychobiological trait^9^ of CO_2_ hypersensitivity^8^, and suggest that both SAD and PD are characterized by a distinctive sensitivity to acidification of brain pH^10^,^11^,^12^, even though individual susceptibility to develop spontaneous panic attacks can typically remain latent throughout childhood and adolescence, and become manifest only in early adulthood. Childhood SAD, CO_2_ hypersensitivity and PD share a high proportion of genes of liability^7^. Moreover, parental separation/loss during childhood heighten the risk for PD, SAD and CO_2_ hypersensitivity^7^, and early-life adversities interact with genetic factors to enhance reactivity to CO_2_-enriched air mixtures^13^. These data indicate a diathesis-stress context, whereby CO_2_ hypersensitivity constitutes an endophenotype of SAD and PD, and early life constitutes a period of vulnerability to develop CO_2_ hypersensitivity in response to adversities via interaction with genetic factors^14^.

In order to model these gene-by-environment interactions (GxE) without the interference of gene-environment correlation, we devised the repeated cross-fostering (RCF), a paradigm of early interference with maternal environment^15^. Our data show that ultrasonic vocalizations (USV, indexing separation anxiety), heightened aversion towards CO_2_-enriched environments, and hyperventilatory responses to 6% CO_2_-enriched air (but not to hypoxic- or normal air conditions) are all significantly augmented among RCF mice, compared to normally-reared animals at 16–20, and 75–90 postnatal days^15^. The specific and stable CO_2_ hypersensitivity induced by RCF has proven reliable and replicable^16^, and is followed by long-term vulnerability to adversities^17^. The RCF procedure is associated with both increased mean and increased variance for tidal volume during 6% CO_2_ breathing^15^; this significant augmentation of genetic variance and heritability of the respiratory responses among RCF animals closely replicate our human findings^13^ of GxE effects in CO_2_ hypersensitivity. The absence of significant differences for maternal cares (nursing, grooming, licking), corticosterone basal levels, or changes in hippocampal mRNA for glucocorticoid/mineralocorticoid receptors between RCF and control animals^15^,^16^ further strengthen a RCF diathesis model of CO_2_ reactivity, and call for molecular investigations of GxE effects in CO_2_ hypersensitivity.

Among the possible mechanisms of GxE are the histonic proteins’ and DNA methylation/acetylation events that affect peri- and post-natal programming of health and disease^18^. Histone modifications in response to early adversities affect gene expression, development, and adaptation to environmental pressure^19^,^20^, with histone deacetylases influencing both health and disease states^21^. However, no study has yet investigated the epigenetic landscape of CO_2_ sensitivity, or its relationships with PD.

Here, we followed the hypothesis that CO_2_ hypersensitivity associated with early-life adversities can be explained by epigenetic mechanisms. Since histone modifications in response to environmental pressure can influence physiology in a durable manner^19^,^20^,^21^, we undertook a genome-wide investigation of altered histone marks in the medulla oblongata (MO) -a major, recognized brain site of CO_2_ chemoception in animal and man^1^,^22^- of outbred mice exposed to the RCF procedure, compared to normally-reared control (CT) animals. In order to index both gene activation and gene silencing, three widely held and reliable histone marks were employed: H3 acetylation of lysine 9 and 14 (H3Ac), trimethylation of H3 lysine 4 (H3K4me3), and trimethylation of lysine 27 (H3K27me3). The first two histone marks are associated with gene activation, and H3K27me3 is associated with gene silencing^23^. While we anticipated that the RCF procedure can affect several phenotypes, our primary lead was the CO_2_ hypersensitivity associated with the RCF procedure. We therefore expected to find evidence of epigenetic modification and altered gene expression compatible with increased sensitivity to acid-base adjustments and the associated pH chemosensory mechanisms.

## Results

### Sequencing and Quality Control

Sequencing ChIP-seq libraries were obtained successfully for H3Ac, H3K4me3, H3K27me3 histone modifications (see Table S1 for details of sequencing performance), thus allowing the comparisons between RCF and CT animals.

**Figure 1:**
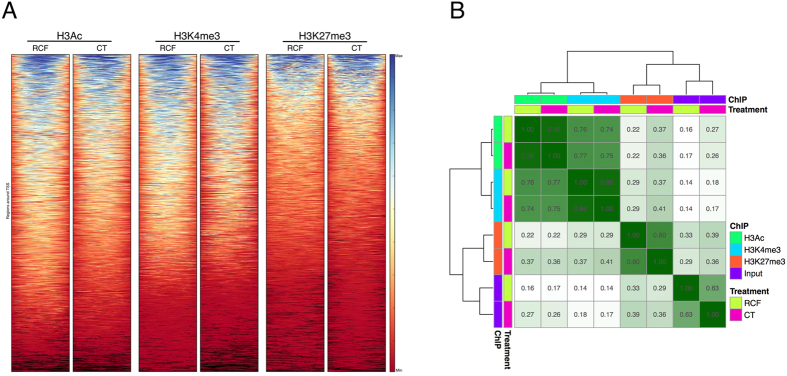
(**A**) Profile heatmap around TSS of RefSeq genes. Read counts were extracted for all ChIP-seq experiments within a region spanning ±5 kb around TSS. The gradient blue-to-red color indicates high-to-low counts in the corresponding region. (**B**) Clustered heatmap of ChIP-seq profiles. Correlations were evaluated over the genome-wide signal of all experiments, including genomic DNA, and shown as a diagonal matrix. Rows and columns are clustered according to inter-sample correlation. Color intensity is proportional to the correlation value reported in each cell. Markers for actively transcribed regions (H3K4me3 and H3Ac) cluster together in the upper left region. H3K27me3 clusters with input DNA, probably due to its broad enrichment profile.

The enrichment profiles of histone marks around the transcriptions start site (TSS, Fig. 1A) yielded sharper patterns of differentiation between RCF and CT for TSS-centered H3Ac and H3K4me3 than for H3K27me3. Correlations among different ChIP-seq profiles calculated on the genome-wide signal (Fig. 1B) were high (0.74 < r < 0.77) between H3K4me3 and H3Ac profiles: this indicates concordance between these two markers of gene activation^21^ and their substantial convergence in reading the epigenomic states. Conversely, H3K27me3 showed higher correlation to input DNA (0.29 < r < 0.36), due to the broad shape of its enrichment profiles^23^.

A survey of the H3K4me3 enrichments among RCF and CT data for the promoter of the 3 MO-enriched genes Esyt1, Glra1 and Prune2, showed as expected 3 peak profiles (Figure S1A); conversely, no such profile was found for a ‘control’ group of 3 MO-unrelated genes: Mef2c, Col6a1 and Neurod6 a (Figure S1B). Under the assumption that H3Ac is associated with actively transcribed genes, this indicates that the tissues used in the experiment were bona fide limited to the medullary region.

### Differential Enrichment Profile in RCF and CT animals

Analyses of differential enrichments for the three markers revealed that the RCF condition was largely associated with enriched regions for the H3Ac and the H3K4me3 histone marks (459 enriched vs. 53 depleted regions, and 232 enriched vs. 12 depleted regions, respectively, Table S2). The H3K27me3 mark, which is functionally linked to repression, was predominantly associated with depletion (572 vs. 439 enriched regions) among RCF animals.

Successive analyses were focused on differential regions proximal to TSS, within a range of −5 kb +1 kb. Since the 3 histone marks employed in our study do not generally act independently^21^, we retained a list of genes that were: a) associated with at least one TSS-proximal mark, and, b) significantly (absolute logFC >1 and p-value < 0.01) enriched/depleted. A total of 1767 regions, corresponding to the proximal promoter region of 1434 genes (Table S3), were retained after application of these criteria.

### SAGE Transcriptome Analyses and Comparison to Epigenomic data

Transcriptome analyses carried out by SAGE on a set (Set 2, see Method section) of MO tissues independent from the set (Set 1, see Method section) employed for ChIP-seq experiments followed, in order to integrate and complement the epigenetic data. Analyses of transcriptomic data showed that out of the 1434 genes yielded by ChIP-seq analyses, 427 were analyzable also by SAGE (Table S4).

Overall, epigenomic and transcriptomic data showed good concordance (Figure S2): fold changes of histone marks associated with gene activation (H3K4me3 and H3Ac) were concordant with fold-changes of corresponding transcripts (respectively, *p*^H3K4me3^ = 1.1E-3, *p*^H3Ac^ = 6.89E-19, by Fisher exact test), and fold-change of H3K27me3 was discordant with transcript level (*p*^H3K27me3^ = 1.01E-57 by Fisher exact test).

### Integrative Analyses

**Figure 2:**
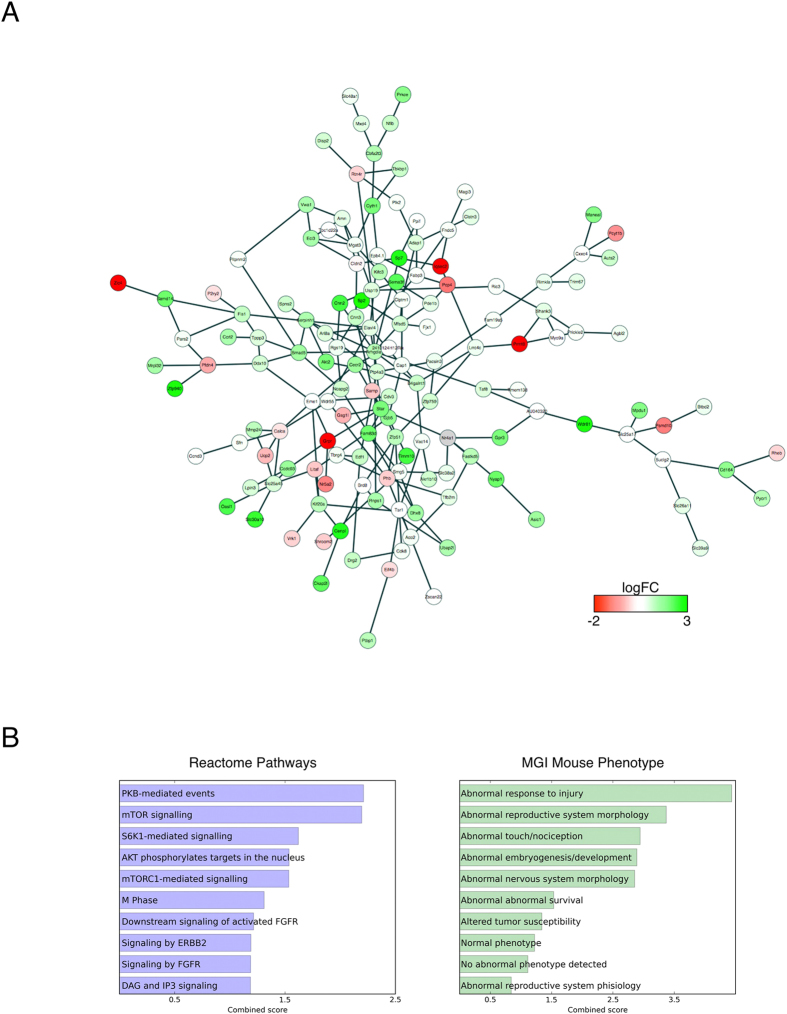
(**A**) Active circuit of 145 genes identified by network analysis (see methods). Each node is colored by the fold change found by SAGE when comparing RCF vs. CT mice. (**B**) Functional analysis of the active circuit. Scores are combined for EnrichR analysis on Reactome Pathways and MGI Mouse Phenotype gene sets.

Analyses of enriched Gene Ontology (GO) categories on the set of 427 genes shared by ChIP-seq and SAGE analyses identified a number of terms linked to neuronal compartments, and supported the ability of the RCF procedure to affect neuronal functions. The successive network analyses (see also methods section) of the previously identified 427 genes, based on co-expression and co-localization data, sought the maximal coalescence of ChIP-seq and SAGE experiments in differentiating RCF from CT animals. The resulting active module (Fig. 2) included 145 genes (Table S5), 13 of which (Table 1) were simultaneously and significantly different between RCF and CT animals for enrichment at the ChIP-seq analyses carried out on MO Set 1, and altered expression at the SAGE analyses carried out on MO Set 2.

Functional enrichment analysis performed with Reactome Pathways and MGI Mouse Phenotype yielded among the top phenotypes encompassed by the 145 genes’ active module network, the following traits: ‘Abnormal Response to Injury’ (encompassing ‘*abnormality of acid-base homeostasis*’ http://monarchinitiative.org/phenotype/HP:0001941, and ‘*increased susceptibility to spontaneous CNS ischemia and decreased resistance to ischemic brain injury*’ http://monarchinitiative.org/phenotype/MP:0003076), ‘Abnormal touch/nociception’ and ‘m- TOR signaling’ both relevant to nociception^24^, and ‘Abnormal nervous system morphology’.

These data (Fig. 2 and Table 1) share a functional substrate of altered acid-base homeostasis associated with the RCF condition, epitomised by the Asic1 gene enrichment and the ASIC1 augmented transcription.

### Real-time PCR analysis

**Figure 3:**
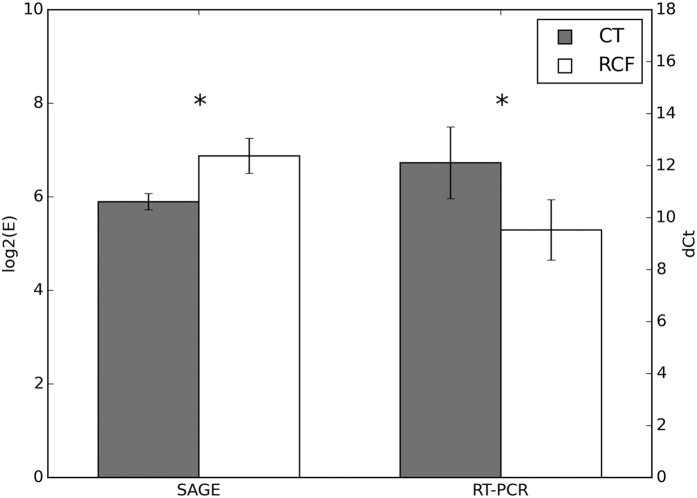
Asic1 gene Expression. Histograms on the left represent mean ± SD levels of expression of Asic1 gene found by SAGE analysis in Set 2 MOs of RCF and CT mice, as described in the main text: mean log 2(E) ± SD Fold Change respectively: 6.88 ± 0.37 and 5.89 ± 0.17, p = 0.047. Histograms on the right represent mean ± SD levels of expression of Asic1 gene found by real time PCR in the MO of 5 RCF and 4 CT mice: mean ± SD of Delta Cycle Threshold (dCT) as referred to Atcb housekeeping gene, respectively: 9.52 ± 1.16 and 12.10 ± 1.38, Student t = 2.64, p = 0.033. Asterisks over the RCF bars denote significant difference in comparison to the CT group.

A real-time PCR analysis of the MOs of 5 RCF and 4 CT 90 days old animals different from those included in the Set 1 and Set 2 tissues, showed significantly increased mRNA expression of the Asic1 gene. These results are shown in the right hand side of Fig. 3, with the left hand side of Fig. 3 focusing on the results of the SAGE experiment specifically for Asic1.

### RCF-Associated Phenotypes

**Figure 4:**
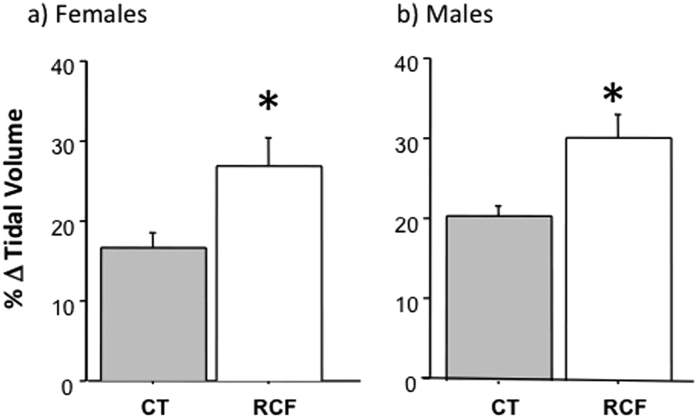
Respiratory responses. Mean ± SD values of the percentage of increment (delta) of tidal volume from baseline (air) to 6% CO_2_-enriched air, among 5 RCF female (26.88 ± 8.10) vs. 5 CT female (16.63 ± 4.12) adult (PND = 80–90) mice (Student t = 2.54, DF = 8, p = 0.03), and 5 RCF male (30.46 + 6.34) vs. 3 CT male (20.68 + 2.02) adult (PND = 80–90) mice (Student t = 2.53, DF = 6, p = 0.05). Asterisk indicates a significance of p ≤ 0.05 in the comparisons of RCF to CT animals divided by sex. The results of an ANOVA with females and males pooled together are reported in the main text.

Prompted by these results we assessed 2 key phenotypes associated with pH variation and ASIC activity, namely respiratory responses to hypercarbia and nociception. We found that RCF animals have a significantly increase of respiratory tidal volume compared to CT animals, when they breathe 6%CO_2_-enriched air mixtures (delta tidal volume respectively: 28.7 ± 2.2 vs. 18.1 ± 1.4; Treatment: F_1,14_ = 12.16, p < 0.01; Sex: F_1,14_ = 1.76, p = ns; interaction Treatment × Sex: F_1,14_ = 0.01, p = ns; see Fig. 4, and caption to Fig. 4 for data divided by sex).

**Figure 5:**
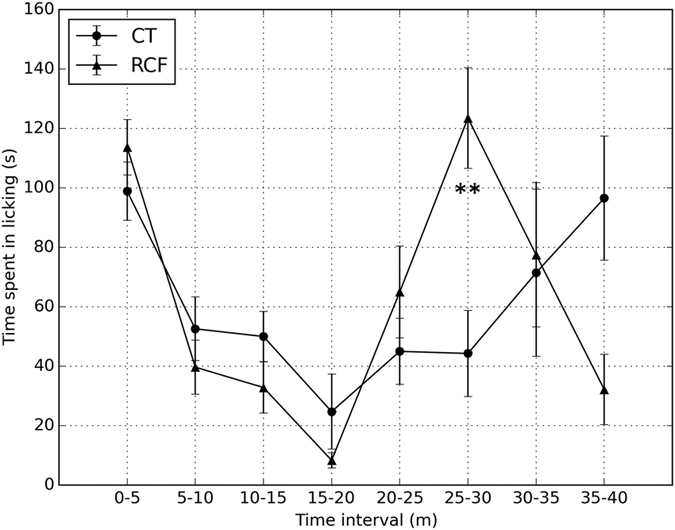
Nociception. Results of the formalin test performed in 6 RCF vs. 7 CT adult (PND = 80–90) male mice. Responses were recorded continuously for 40 minutes and calculated in blocks of consecutive 5 minute periods. The figure shows the time spent licking the injected paw (Mean ± SE in seconds) for each time interval; ‘*’ and ‘**’ indicate significance of respectively p < 0.005 and p < 0.0001 when comparing RCF to CT animals at specific time intervals.

We also found significantly altered nociception associated with the RCF paradigm (Fig. 5). A time (ANOVA F1, 7 = 5.8, p < 0.0001) and time-by-treatment interaction (F1, 77 = 3.2, p < 0.005) effect at the formalin test indicated significantly stronger reaction to a painful stimulus among RCF compared to CT animals during the tonic phase of response to pain.

## Discussion

Our data show an association between the RCF procedure -a paradigm of early interference with maternal environment- and histone marks of extensive enrichment in the medulla oblongata, a major site of chemoreception, and hence of respiratory control. Conjoint functional enrichment and RNA transcript analyses yielded a coherent picture for genes affecting chemoreception and nociception –*i.e*. Asic1-, and neurodevelopment. The enrichment of Asic1 is concurrently coherent with the presence of: Abnormal Response to Injury’ (encompassing ‘abnormality of acid-base homeostasis’ ‘increased susceptibility to spontaneous CNS ischemia and decreased resistance to ischemic brain injury’), ‘Abnormal touch/nociception’ and ‘m-TOR signaling’, among the top phenotypes associated with the RCF procedure. All these 3 phenotypes share the fundamental feature of being linked to acidosis, a state readily detected by the ASIC channels that underlies variation in both CO_2_ sensitivity and nociception^25^. Consistent with this, and in keeping with our main hypothesis, RCF mice showed both exaggerated respiratory response to hypercapnia and altered nociception, compared to CT animals. The ASIC1a ion channels for which Asic1 codifies are widely expressed in the brain including the MO^25^, and are pivotal in driving acidosis-sensing mechanisms and in mediating amygdala-originated fear in response to CO_2_^4^. A study with Asic1−/− animals, however, showed unaltered responses to hypercapnia^4^. This counterintuitive finding could nonetheless be connected to compensatory mechanisms during development^4^. Coherently with our findings, the human ortholog of the Asic1 gene, ACCN2, has been found associated with PD and with amygdala function in a multiple cohort study^26^, and ACCN2 genetic variants have been found associated with CO_2_ hypersensitivity in humans^27^.

Turning to altered nociception, early adversities including maternal separation are associated with diminished nociceptive threshold/hyperalgesia^28^ in both rats and mice. In humans, the occurrence of early adversities followed later in life by comorbid anxiety and pathological pain syndromes (exaggerated/aberrant nociception) provide a staggering, and yet poorly understood case for comorbidity. Large-scale rigorous general population epidemiological surveys found associations between chronic pain syndromes (including arthritis, back pain, migraine) and panic and generalised anxiety disorders with odds ratios (2.5–3.2) that outnumbered the average odds ratio (2.1) for association between pain and depression^29^. Moreover, the association between panic anxiety and pain syndromes was more recently confirmed by the National Comorbidity Replication Study results^30^.

Several other genes, mostly affecting neurodevelopment, were differentially enriched and differentially-expressed following the RCF procedure (Table 1). Their known functions include widespread synaptic plasticity and organization (Cbln1)^31^, hippocampus’ and cortical development, learning and fear (Bmpr1b)^32^,^33^, development of olfactory bulb and hippocampus (Tsc22d4)^34^, hippocampal neurogenesis (Grpr)^35^, integration of stress responses (Grpr)^36^ and mother-pup attachment by odour recognition (Grpr)^37^. Further neurodevelopmental functions subserved by enriched and differentially-expressed genes in RCF animals include neuronal survival (Wdr81)^38^ and development of neural stem cells into mature cortical and olfactory bulb neurons (Sp2)^39^.

The bulk of these data indicate neurodevelopment and neurogenesis as further phenotypes that become possibly altered following the RCF procedure, and merit further investigation that may extend beyond the MO, including the amygdala and the bed nucleus of the stria terminalis^40^.

When looking at these data, some limitations are also in order. While our investigation was primed by the well-documented ability of the RCF procedure to induce CO_2_ hypersensitivity, the associations of epigenetic changes shown in this paper are with RCF, not necessarily with CO_2_ response or altered nociception. Additional work is thus needed to clarify and confirm the full fraction of the epigenetic response involved in CO_2_ hypersensitivity, and which genes are epigenetically programmed as part of the comprehensive response to RCF.

Also, epigenetic changes seen in adulthood may constitute a downstream consequence of the phenotype(s), rather than the original alterations that elicited the phenotype(s): further experiments, including longitudinal assessments and manipulations of candidate genes, that test in a more cogent way the causal pathways between the genes identified here and CO_2_ response and altered nociception will clarify this point.

While the RCF-associated phenotype of CO_2_ hypersensitivity constitutes a non-inferential translational model of human PD, ours remains a reductionist approach; it should be remembered that panic attacks present with various physical and cognitive symptoms beyond hyperventilation, and hypercarbia induces several other behavioural and physiological responses such as freezing^4^, increased blood pressure, or tachycardia. As far as humans are implied, some consider the link between responses to CO_2_ challenges and the SAD-PD-AGO continuum relatively non-specific (see^14^ for a reappraisal of this issue), an argument in this connection being the putative role of third, ‘latent variables’ such as ‘general anxiety factors’ (including baseline anxiety, or anxiety sensitivity)^41^ as possible mediators of the response to hypercarbia in humans. From a genetically-informed vantage point, a compelling assessment of the relevance of this claim consists in testing whether and to what extent the polygenic background of CO_2_–evoked anxious responses overlaps with the genetic background of ‘trait’, or ‘general’ anxiety. Our^42^ empirical treatment of this issue showed that in man there are distinct genetic factors associated with responsiveness to respiratory stimulation via 35% CO_2_, as compared to the genetic factors that influence baseline, pre-CO_2_ anxiety influences^42^.

Within the limitations of this specific experimental approach, our findings yield 4 main points of broader relevance. First, histone modifications and the correlated, consistent variations of expression profiles among RCF animals provide a molecular basis for a GxE effect which we had previously identified by quantitative genetic approaches to explain CO_2_ hypersensitivity in man^13^ and animal^15^. In the laboratory animal context we were able to exclude the gene-environment correlations that almost invariably hamper human gene- environment interaction research. Second, intermediate phenotypes of physiological nature – including respiratory responses to CO_2_ for PD/SAD- facilitate parallel investigations in man and animal. By this strategy, we could drive our focus onto specific physiological functions and phenotypes characteristic of RCF-exposed animals, and identify the pertinence of their implication in human PD. Third, by a developmental approach, the relevance of genetic, environmental factors and the resulting epigenetic modifications to explain biological variation and pathophysiology emerge more precisely. Fourth, inasmuch as the relationships between peripheral- and brain measures of epigenetic changes are emerging more clearly^43^, epigenetic marks can constitute a further element of risk prediction for human psychopathology.

## Methods

### Animals

NMRI outbred mice (Harlan, Italy) were used in all experiments. Mice were mated when they were 12 weeks old. Mating protocol consisted in housing two females with one male for 15 days in transparent high temperature polysufone cages (26.7 × 20.7 × 14.0 cm) with water and food available *ad libitum*. Room temperature (21 ± 1 °C) and a 12:12 h light dark cycle (lights on at 07.00 a.m.) were kept constant. Pregnant females were isolated in clean cages, and inspected twice a day for live pups. For the first postnatal day (PND0) litters were left with the biological mother. All experiments were conducted in accordance with the guidelines approved by the Italian Health Department, in accordance with Italian regulations on the ethical use of research animals (legislationDL 116/92), and the NIH guidelines on animal care. All experimental protocols had been approved by the ethical committee of the National Research Council.

### RCF procedure

The RCF is a cross-fostering procedure originally devised to interfere with infant-mother relations in the first days of life, so to elicit offspring’s separation anxiety without inducing neglect from caregivers^13^. The RCF procedure has been fully detailed elsewhere^15^,^16^,^17^. Briefly, having spent the first postnatal day (PND0) with the biological mother, on PND1 litters were culled to 8 pups (50% females) and assigned to RCF, or control (CT) treatment. Unlike more ‘classical’ cross- fostering procedures, RCF pups changed caregiver every 24 hours for 4 consecutive times in the PND1-PND4 time interval. By following a rotation scheme, each dam was shifted to 4 different litters and each litter was shifted to 4 different dams. The procedure of removing and cross- shifting a mother and the litter lasted about 30 seconds and was repeated daily, 4 times (PND1 to PND4), until reaching the fourth adoptive mother, with which pups remained until weaning. Adoptive dams were lactating females with pups of the same age as the fostered litters. Control litters were collected daily and reintroduced to their home-cage, and had their biological mothers returned within 30 sec, from PND1 to PND4. Animals were weaned when 28 days old, and then separated by sex and left in cages with littermates until sacrificed. No significant sex effects were found for the RCF-evoked respiratory phenotypes in this data set (see Fig. 4) as well as in our previous work^14^,^15^,^16^. We used females in all the epigenetic experiments because the epigenetic profile is known to vary in the two sexes^20^,^23^, and because PD, the human mental disorder to which the RCF protocol is referred, is more common in women^6^,^8^. Since different forms of environmental pressure/stress can induce histone modifications^19^,^20^, all the tissues employed in this study came from animals that had not been exposed to CO_2_ respiratory probing, or nociceptive tests. This ensured that none of the changes in the epigenetic or expression profiles we observed could be attributable to other procedures except the RCF.

### Samples for chromatin immunoprecipitation and gene expression analyses

Brain stems were collected from 90 days old females belonging to 5 RCF and 5 CT litters (2 animals/litter), with one of us (RC) performing all the dissections for the sake of homogeneity. In order to ensure sufficient amounts of tissues, a first set of MOs (Set 1, one animal/litter: 5 RCF and 5CT mice) was employed for chromatin immunoprecipitation, and the second set (Set 2, one animal/litter: 5 RCF and 5CT mice) was employed for serial analysis of gene expression (SAGE) (see below).

### Synoptic Presentation of Methods for Epigenetic Analyses

A synoptic presentation of the methods and statistics employed for the epigenetic analyses can be found in Figure S3.

### Chromatin immunoprecipitation (ChIP) and library preparation

Tissues from Set 1 were homogenized, nuclei were prepared, and chromatin was partially digested by micrococcal nuclease^44^. Equal amounts of digested chromatin were used to perform chromatin immunoprecipitation (ChIP) with antibody against histone H3 acetylation of lysine 9 and 14 (H3Ac; Millipore #06–599), histone H3 lysine 4 trimethylation (H3K4me3; Millipore #17–614), and histone H3 lysine 27 trimethylation (H3K27me3; Millipore #17–622). ChIP DNA was then used to obtain sequencing-ready libraries for the SOLID sequencing platform (Applied Biosystems, Waltham, USA). Libraries were also obtained and sequenced from the Input DNA to which no ChIP procedure was applied.

### ChIP-seq analysis

Short reads were aligned to mouse reference genome (mm9) using Color Space format via Life Technologies Lifescope 2.5.1 analysis software. After removal of duplicated reads, we identified ChIP enrichments using MACS2^45^. Enrichment profiles at TSS were obtained with Fluff package (http://github.com/simonvh/fluff). Correlation among enrichment profiles was calculated with the wigCorrelate tool, which is part of UCSC genome browser utilities. We then applied an Irreproducible Discovery Rate (IDR) procedure^46^ by selecting consistent peaks over an IDR threshold of 540 (p < 0.05) after splitting each dataset into 2 pseudo-replicates, according to IDR best practices. Raw read counts were extracted from every resulting region. These counts were then analysed using edgeR^47^. Trimmed Mean of M-values (TMM) normalization was used to equalize library sizes. Pairwise comparisons were made by exact negative binomial tests. Differential peaks were defined under a p-value threshold of 0.01 and log-FoldChange threshold of ±1.

Peaks were annotated using the RefSeq transcript definition^48^, and were assigned to a gene if they overlapped of an interval of −5 kb + 1 kb from the transcriptions start site (TSS).

### SAGE analysis

Total RNA was extracted individually from one set (Set 2) of dissected brain stems using a Total RNA Purification Plus kit (Norgen Biotek, Thorold, ON, Canada), after homogenization in Lysis Buffer with a glass-glass Dounce homogenizer. RNA quantity was measured by absorbance at 260 nm using a NanoDrop UV-VIS spectrophotometer (Thermo Fisher Scientific, Wilmington, DE, USA). The quality and integrity of each sample were confirmed using a BioAnalyzer 2100 (RNA 6000 Nanokit; Agilent, Santa Clara, CA, USA); all samples showed an RNA Integrity Number higher than 7.0.

SAGE barcoded libraries were prepared from total RNA samples. Each library was generated by the SOLiDTM SAGETM kit with the Barcoding Adaptor Module, and a SOLiDTM RNA Barcoding kit (Catalog nos 4452811 and 4427046, Life Technology), by following the manufacturer’s instructions. The barcoded libraries were combined and sequenced on 3 full slides on an Applied Biosystems SOLiD 4 System. Sequencing length was 35 bp. Library preparation, barcode addition, emulsion PCR, and SOLiD sequencing were performed at Genomnia Srl (Lainate, Milan, Italy). Table S6 summarizes the samples and the target mapping percentages associated with this experiment.

All the SAGE Color Space sequence files generated by the sequencing were corrected *de novo* for sequencing errors before mapping on the mouse reference transcriptome, by the Life Technologies SAET (SOLiD Accuracy Enhancer Tool) version 2.2 program (https://www.biostars.org/static/downloads/solid/solid-denovo- assembly/saet.2.2/SAET.v2.2.pdf).

Tag counts were normalized with TMM and analyzed in limma^49^. In order to reduce individual variability associated with having 5 RCF and 5 CT outbred animals, and improve signal over noise, we allowed for 5 latent variables by the R/Bioconductor sva package into the linear model that contrasted the differential expression between RCF vs. CT mice.

### Concordance between ChIP and SAGE data

The concordances between ChIP-seq and SAGE data were tested by the Fisher test applied to the genes’ expected vs. observed direction of fold change (scatterplot depicted in Figure S2). For markers of activation (H3K4me3 and H3Ac) genes are expected to appear in the 1st (++) and 3rd (−−) quadrant, and for H3K27me3 genes are expected to appear in the 2nd (−+) and 4th (+−) quadrant.

### Graph analysis

Genes associated with a differential peak in their TSS region according to the criteria explained above were employed to build a functional graph based on 4 co-expression and co-localization networks available in GeneMANIA^50^, namely the Akahoshi-Ishii-2011, Siddiqui-Marra-2005, Thorrez-Schuit-2008 and Zapala-Barlow-2005.The resulting graph was processed with jActiveModules^51^ by weighting every node with the p-value resulting from ChIP-seq and SAGE experiments. Missing data (i.e. genes not assessed by SAGE or by any of the histone marks) were assigned a p value equal to 1.

Functional enrichment analysis on genes included in the active component was performed by the Enrichr platform^52^.

### Real-time PCR analysis

Ninety days old animals (5 RCF and 4 CT) different from those included in the Set 1 or Set 2 tissues were sacrificed and brains were rapidly removed and placed onto an ice-cooled metal plate. Brainstems were dissected, the MO isolated, and samples were immediately frozen on dry ice and stored at −80 °C. RNA was extracted from homogenized MOs with a Total RNA purification kit (Norgen Biotek, Thorold, ON, Canada) following the instructions of the manufacturer. RNA quantity was determined by absorbance at 260 nm using a NanoDrop UV-VIS spectrophotometer (Thermo Fisher Scientific, Wilmington, DE, USA). RNA was reverse-transcribed with a High-Capacity cDNA Reverse Transcription Kit (Applied Biosystem, Paisley, UK) according to the manufacturer’s instructions. Equal amounts of cDNA were then real-time PCR analysed with an Applied Biosystems7900HT thermal cycler, using the SensiMixSYBR Kit (Bioline, London, UK) and specific primers for Asic1 gene (sense: TTTGTGTCTTGCCAGGAGCAG; antisense: TGGTAACAGCATTGCAGGTG) at a final concentration of 200 nM. The measurement was performed in quadruplicate and the experiment in triplicate. The expression data were normalized using the expression values of the Actb gene. Amplification efficiency for primer pairs was determined by amplification of a linear standard curve (from 0.1 ng to 20 ng) of total cDNA as assessed by A260 spectrophotometry. Standard curves displayed good linearity and amplification efficiency for all primer pairs.

### RCF-Associated Phenotypes

Assessment of the association between the RCF procedure and respiratory hypersensitivity to CO_2_ was attained by measuring the changes in tidal volume during 6% CO_2_-enriched air breathing (CO_2_ challenge) of 5 female +5 male adult RCF, vs. 5 female +3 male CT adult (PND = 80–90) animals in an unrestrained plethysmograph (PLY4211, Buxco Electronics, Sharon CT), as described in details elsewhere^15^,^16^. Briefly, before any recording, each animal was acclimatized for 40 minutes. Then, the recording of respiratory parameters started under air condition (baseline) for 20 minutes. Next, the challenge began with the administration of 6% CO_2_ enriched air for 20 minutes, followed by a 20 minutes recovery period (air). Percentage of increment of tidal volume from the 20 minutes baseline air condition (ΔTV%) to 20 minutes 6% CO_2_ stimulation^15^ were compared between RCF vs. CT animals by two-way ANOVA (factors: treatment and sex), and by Student t test.

A formalin test was used to evaluate the response to inflammatory pain in 6 RCF vs. 7 CT adult (PND = 80–90) male mice. Briefly, one animal at a time was put in standard Plexiglas observational cages of 30 × 12 × 13 cm. Successively, 20 μl of 5% formalin saline solution were injected subcutaneously into the dorsal surface of the right paw by a microsyringe with a 26-gauge needle. Mice were then put back into the chamber with a mirror and the observation period started. The total amount of time spent licking the injected paw was taken as index of pain and measured in seconds. Responses were video-recorded in continuous for 40 minutes, calculated in blocks of consecutive 5-minute periods, and scored by an observer blind to the animal’s status (RCF, or CT). Differences in nociceptive responses between RCF and CT animals were compared by 2-way repeated measure ANOVA (factors: treatment and time).

## Additional Information

**How to cite this article**: Cittaro, D. *et al*. Histone Modifications in a Mouse Model of Early Adversities and Panic Disorder: Role for *Asic1* and Neurodevelopmental Genes. *Sci. Rep*. **6**, 25131; doi: 10.1038/srep25131 (2016).

## Supplementary Material

Supplementary Information

## Figures and Tables

**Table 1:** List of 13 genes that are simultaneously associated with at least one significantly different peak in their TSS-proximal region by ChIP-Seq analyses in MO Set 1, and differentially expressed in RCF vs CT contrast, according to SAGE analysis of MO Set 2.

Gene Symbol	Description
Cbln1	Cerebellin 1 Precursor
Rad51b	DNA Repair Protein RAD51 Homolog 2
E130006D01Rik	lincRNA
Bmpr1b	Bone morphogenetic protein receptor, type 1B
Tsc22d4	TSC22 domain family, member 4
Alg8	Asparagine-linked glycosylation 8
Grpr	Gastrin releasing peptide receptor
Asic1	Acid Sensing Channel 1
Myadm	Myeloid-associated differentiation marker
Cdh26	Cadherin-like 26
Sp7	Sp7 transcription factor 7
Wdr81	WD repeat domain 81
Sp2	Sp2 transcription factor
