# Supplementary material for: Histone Modifications in a Mouse Model of Early Adversities and Panic Disorder: Role for Asic1 and Neurodevelopmental Genes
Source: Sci Rep. 2016 Apr 28;6:25131. doi: 10.1038/srep25131 (PMC4848503; doi:10.1038/srep25131)
Supplement: Supplementary Information [file srep25131-s1.pdf]

Supplementary information for "Histone Modifications in a Mouse Model of Early Adversities and Panic Disorder: Role for *Asic1* and Neurodevelopmental Genes"

Davide Cittaro, Valentina Lampis, Alessandra Luchetti, Roberto Coccurello, Alessandro Guffanti, Armando Felsani, Anna Moles, Elia Stupka, Francesca R. D'Amato and Marco Battaglia

Figure S1: Multiple ChIP-seq profiles at promoters of genes considered positive (A) and negative controls (B) for medulla oblongata tissue. For each gene, the expression levels in different brain sections is reported according to the Allen Brain Atlas.

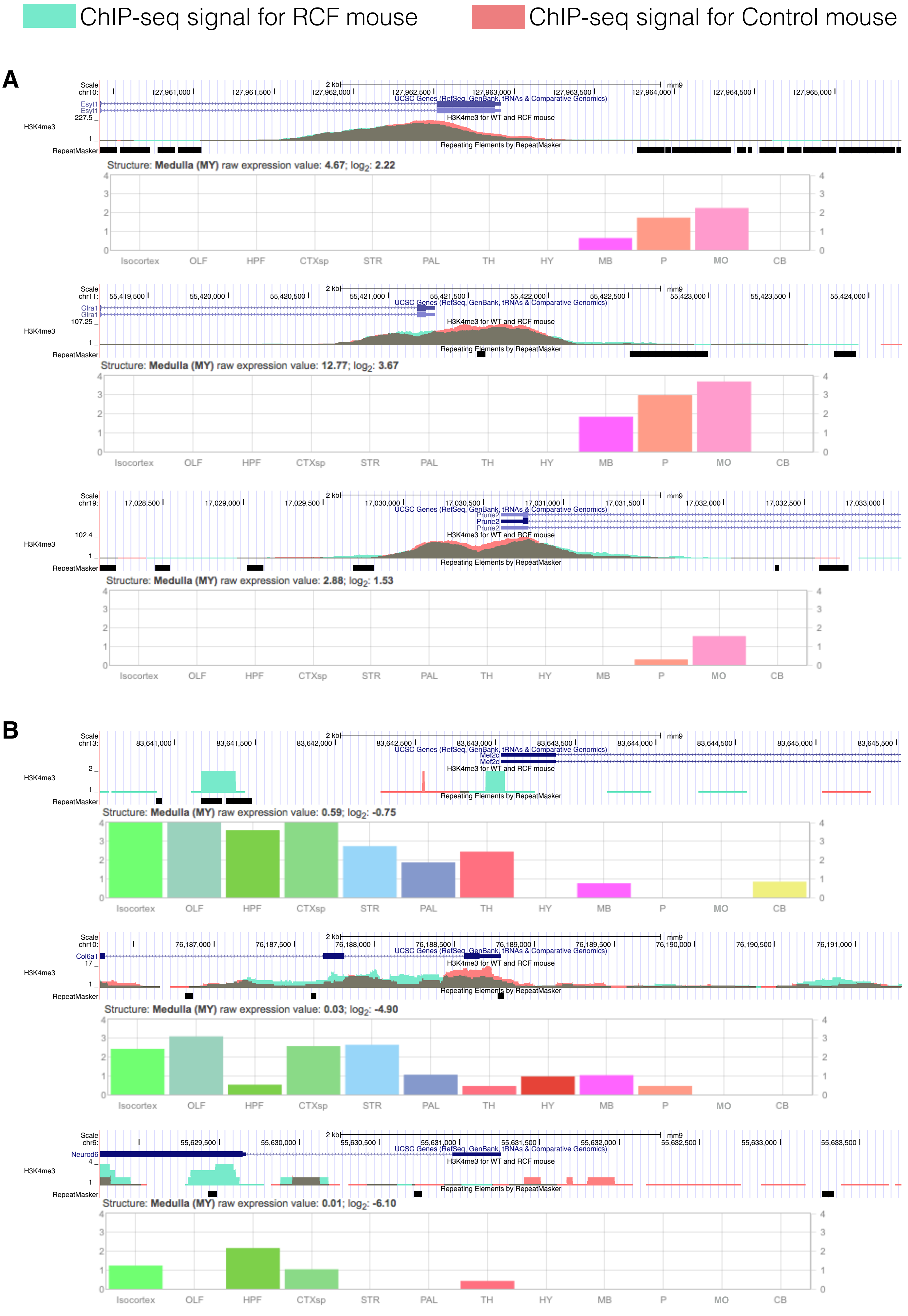

ChIP-seq signal for RCF mouse
ChIP-seq signal for Control mouse
A
Scale
chr10:
Esyt1
H3K4me3
RepeatMasker
UCSC Genes (RefSeq, GenBank, tRNAs & Comparative Genomics)
H3K4me3 for WT and RCF mouse
Repeating Elements by RepeatMasker
Structure: Medulla (MY) raw expression value: 4.67; log2: 2.22
Isocortex
OLF
HPF
CTXsp
STR
PAL
TH
HY
MB
P
MO
CB
chr11:
Glra1
Structure: Medulla (MY) raw expression value: 12.77; log2: 3.67
chr19:
Prune2
Structure: Medulla (MY) raw expression value: 2.88; log2: 1.53
B
chr13:
Mef2c
Structure: Medulla (MY) raw expression value: 0.59; log2: -0.75
chr10:
Col6a1
Structure: Medulla (MY) raw expression value: 0.03; log2: -4.90
chr6:
Neurod6
Structure: Medulla (MY) raw expression value: 0.01; log2: -6.10

Figure S2: Scatterplot showing the relationship between fold change of significant peaks in all ChIP-seq experiments and the fold change of the corresponding transcript level according to SAGE. Labeled genes were simultaneously and significantly different between RCF and CT animals for enrichment at the ChIP-seq analyses and altered expression at the SAGE

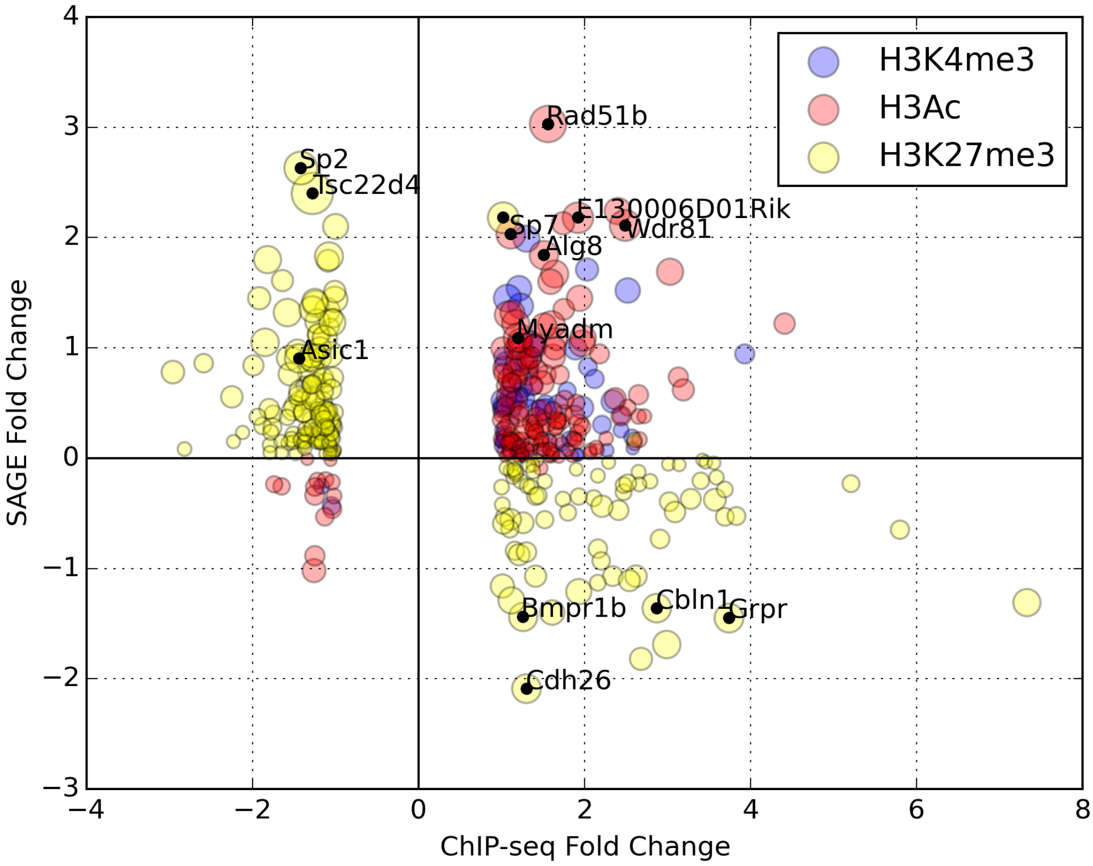

Figure S3: Synopsis of methods employed for the epigenetic analyses

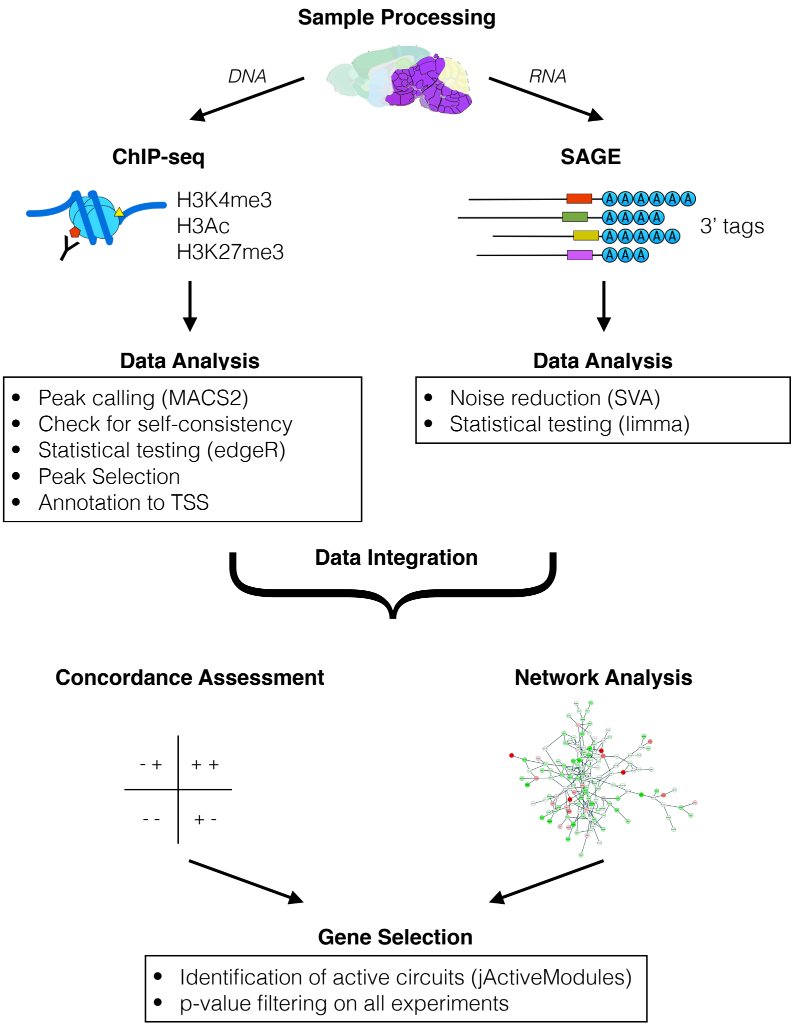

Medulla oblongata tissue set 1

Peaks were identified using MACS2. Self-consistent peaks were selected by Irreproducible Discovery Rate (IDR < 0.05). Differential enrichments in RCF vs CT were assessed by negative binomial test implemented in edgeR. Differentially enriched peaks (p < 0.01, |logFC|>1) were annotated to the TSS of RefSeq genes, within an interval of -5kb +1kb

Concordances between ChIP-seq and SAGE data were tested by the Fisher test applied to fold-change location.

Medulla oblongata tissue set 2

RCF vs CT tag counts were compared by a linear model with five latent variables extracted with SVA to account for individual variation.

Network analysis was performed on a set of 427 genes simultaneously accounted for by ChIP-seq and SAGE analyses. Every node was weighted with the p-value resulting from ChIP-seq and SAGE experiments.

Successive network analyses were based co-expression and co-localization data; they yielded an active module of 145 genes, 13 of which were simultaneously and significantly different between RCF and CT animals for enrichment at the ChIP-seq analyses and altered expression at the SAGE

Table S1: Number of reads recovered for each ChIP-seq experiment

Marker	Condition	Total reads
H3Ac	CT	17127549
H3K27me3	CT	12136204
H3K4me3	CT	10877097
Control	CT	27182302
H3Ac	RCF	21085548
H3K27me3	RCF	17818983
H3K4me3	RCF	12717825
Control	RCF	27784026

Table S2: Counts of TSS-proximal peaks (-5kb+1kb) that are significantly different in enrichment comparing RCF to CT mice.

	H3K4me3	H3Ac	H3K27me3
**Enriched**	232	459	439
**Depleted**	12	53	572
**Total**	**244**	**512**	**1011**

Table S3: List of genes associated with at least one significantly different peak in any ChIP-seq experiment in their TSS-proximal region when comparing RCF to CT mice.

MGI symbol	Entrez ID	Description
**0610031J06Rik**	.	.
**0610040B10Rik**	67672	RIKEN cDNA 0610040B10 gene
**0610040B10Rik**	.	.
**1600029I14Rik**	69797	RIKEN cDNA 1600029I14 gene
**1700003M07Rik**	72225	RIKEN cDNA 1700003M07 gene
**1700003M07Rik**	.	.
**1700011A15Rik**	66322	RIKEN cDNA 1700011A15 gene
**1700012D01Rik**	72243	RIKEN cDNA 1700012D01 gene
**1700019O17Rik**	71863	RIKEN cDNA 1700019O17 gene
**1700021N21Rik**	.	.
**1700021N21Rik**	69409	RIKEN cDNA 1700021N21 gene
**1700025G04Rik**	69399	RIKEN cDNA 1700025G04 gene
**1700028D13Rik**	.	.
**1700028D13Rik**	75569	RIKEN cDNA 1700028D13 gene
**1700029F12Rik**	66479	RIKEN cDNA 1700029F12 gene
**1700029M20Rik**	73937	RIKEN cDNA 1700029M20 gene
**1700029M20Rik**	.	.
**1700031F05Rik**	73300	RIKEN cDNA 1700031F05 gene
**1700034H15Rik**	98736	RIKEN cDNA 1700034H15 gene
**1700034H15Rik**	.	.
**1700044C05Rik**	.	.
**1700044C05Rik**	73306	RIKEN cDNA 1700044C05 gene
**1700084C01Rik**	78465	RIKEN cDNA 1700084C01 gene
**1700102P08Rik**	112418	RIKEN cDNA 1700102P08 gene
**1700113H08Rik**	76640	RIKEN cDNA 1700113H08 gene
**1700125G02Rik**	.	.
**1700125G02Rik**	73628	RIKEN cDNA 1700125G02 gene
**1810009A15Rik**	66276	RIKEN cDNA 1810009A15 gene
**1810013A23Rik**	69050	RIKEN cDNA 1810013A23 gene
**1810044D09Rik**	.	.
**1810044D09Rik**	69798	RIKEN cDNA 1810044D09 gene
**2310036O22Rik**	68544	RIKEN cDNA 2310036O22 gene
**2310045N01Rik**	72368	RIKEN cDNA 2310045N01 gene
**2410004I01Rik**	.	.
**2410004I01Rik**	69739	RIKEN cDNA 2410004I01 gene
**2410016O06Rik**	71952	RIKEN cDNA 2410016O06 gene
**2410124H12Rik**	.	.
**2410124H12Rik**	76785	RIKEN cDNA 2410124H12 gene

**2610002J02Rik**	.	.
**2610028E06Rik**	72395	RIKEN cDNA 2610028E06 gene
**2610028E06Rik**	.	.
**2610206C17Rik**	.	.
**2610206C17Rik**	72495	RIKEN cDNA 2610206C17 gene
**2610306M01Rik**	67170	RIKEN cDNA 2610306M01 gene
**2610306M01Rik**	.	.
**2810408I11Rik**	.	.
**2810408I11Rik**	69941	RIKEN cDNA 2810408I11 gene
**2900076A07Rik**	.	.
**2900076A07Rik**	100504421	RIKEN cDNA 2900076A07 gene
**3110007F17Rik**	.	.
**3110009E18Rik**	73103	RIKEN cDNA 3110009E18 gene
**3200001D21Rik**	71818	RIKEN cDNA 3200001D21 gene
**4631405J19Rik**	433456	RIKEN cDNA 4631405J19 gene
**4631405J19Rik**	.	.
**4732416N19Rik**	320737	RIKEN cDNA 4732416N19 gene
**4732491K20Rik**	.	.
**4732491K20Rik**	224523	RIKEN cDNA 4732491K20 gene
**4833420G17Rik**	67392	RIKEN cDNA 4833420G17 gene
**4921504E06Rik**	70909	RIKEN cDNA 4921504E06 gene
**4921509O07Rik**	70917	RIKEN cDNA 4921509O07 gene
**4921513I03Rik**	.	.
**4921513I03Rik**	70874	RIKEN cDNA 4921513I03 gene
**4921530L21Rik**	66732	RIKEN cDNA 4921530L21 gene
**4930413E15Rik**	74627	RIKEN cDNA 4930413E15 gene
**4930432J09Rik**	73877	RIKEN cDNA 4930432J09 gene
**4930455H04Rik**	.	.
**4930455H04Rik**	74654	RIKEN cDNA 4930455H04 gene
**4930470P17Rik**	.	.
**4930470P17Rik**	67637	RIKEN cDNA 4930470P17 gene
**4930471M09Rik**	.	.
**4930471M09Rik**	75787	RIKEN cDNA 4930471M09 gene
**4930483J18Rik**	.	.
**4930483J18Rik**	67638	RIKEN cDNA 4930483J18 gene
**4930483O08Rik**	74970	RIKEN cDNA 4930483O08 gene
**4930500F04Rik**	70969	RIKEN cDNA 4930500F04 gene
**4930519F16Rik**	.	.
**4930519F16Rik**	75106	RIKEN cDNA 4930519F16 gene
**4930522H14Rik**	67646	RIKEN cDNA 4930522H14 gene
**4930543E12Rik**	75239	RIKEN cDNA 4930543E12 gene
**4930570G19Rik**	.	.
**4930570G19Rik**	329782	RIKEN cDNA 4930570G19 gene

**4931428F04Rik**	74356	RIKEN cDNA 4931428F04 gene
**4933406K04Rik**	.	.
**4933406K04Rik**	71033	RIKEN cDNA 4933406K04 gene
**4933407E24Rik**	108809	RIKEN cDNA 4933407E24 gene
**4933407E24Rik**	.	.
**4933416E03Rik**	.	.
**4933416E03Rik**	71081	RIKEN cDNA 4933416E03 gene
**4933417O13Rik**	71153	RIKEN cDNA 4933417O13 gene
**4933417O13Rik**	.	.
**4933428G20Rik**	100141474	RIKEN cDNA 4933428G20 gene
**4933432K03Rik**	75793	RIKEN cDNA 4933432K03 gene
**4933433G15Rik**	71274	RIKEN cDNA 4933433G15 gene
**4933433G15Rik**	.	.
**4933434E20Rik**	99650	RIKEN cDNA 4933434E20 gene
**4933439K11Rik**	71319	RIKEN cDNA 4933439K11 gene
**4933439K11Rik**	.	.
**5530601H04Rik**	71445	RIKEN cDNA 5530601H04 gene
**5530601H04Rik**	.	.
**5730408K05Rik**	67531	RIKEN cDNA 5730408K05 gene
**5830416I19Rik**	74757	RIKEN cDNA 5830416I19 gene
**6030408B16Rik**	.	.
**6030408B16Rik**	77717	RIKEN cDNA 6030408B16 gene
**6530411M01Rik**	.	.
**6530411M01Rik**	67791	RIKEN cDNA 6530411M01 gene
**8430408G22Rik**	213393	RIKEN cDNA 8430408G22 gene
**9130023H24Rik**	100043133	RIKEN cDNA 9130023H24 gene
**9230105E05Rik**	.	.
**9230105E05Rik**	320626	RIKEN cDNA 9230105E05 gene
**9330133O14Rik**	100689703	RIKEN cDNA 9330133O14 gene
**9330133O14Rik**	.	.
**9430038I01Rik**	77252	RIKEN cDNA 9430038I01 gene
**9530080O11Rik**	.	.
**9530080O11Rik**	319247	RIKEN cDNA 9530080O11 gene
**A130010J15Rik**	319266	RIKEN cDNA A130010J15 gene
**A230056J06Rik**	319469	RIKEN cDNA A230056J06 gene
**A330041J22Rik**	319556	RIKEN cDNA A330041J22 gene
**A330041J22Rik**	.	.
**A330069E16Rik**	.	.
**A330069E16Rik**	606735	RIKEN cDNA A330069E16 gene
**A330070K13Rik**	381673	RIKEN cDNA A330070K13 gene
**A630019I02Rik**	408254	RIKEN cDNA A630019I02 gene
**A630075F10Rik**	.	.
**A630075F10Rik**	100043910	RIKEN cDNA A630075F10 gene

**A730018C14Rik**	100504733	RIKEN cDNA A730018C14 gene
**A730043L09Rik**	330958	RIKEN cDNA A730043L09 gene
**AU015836**	385493	expressed sequence AU015836
**AU015836**	.	.
**AU040320**	100317	expressed sequence AU040320
**Aak1**	269774	AP2 associated kinase 1
**Aanat**	11298	arylalkylamine N-acetyltransferase
**Abcf3**	27406	ATP-binding cassette, sub-family F (GCN20), member 3
**Abhd4**	105501	abhydrolase domain containing 4
**Abi3**	66610	ABI gene family, member 3
**Acn9**	.	.
**Aco2**	11429	aconitase 2, mitochondrial
**Acot11**	329910	acyl-CoA thioesterase 11
**Acot13**	66834	acyl-CoA thioesterase 13
**Acox3**	80911	acyl-Coenzyme A oxidase 3, pristanoyl
**Actl10**	70362	actin-like 10
**Actrt2**	73353	actin-related protein T2
**Adam11**	11488	a disintegrin and metallopeptidase domain 11
**Adam33**	110751	a disintegrin and metallopeptidase domain 33
**Adap1**	231821	ArfGAP with dual PH domains 1
**Add2**	11519	adducin 2 (beta)
**Adrbk2**	320129	adrenergic receptor kinase, beta 2
**Agbl2**	271813	ATP/GTP binding protein-like 2
**Agmat**	75986	agmatine ureohydrolase (agmatinase)
**Ago2**	239528	argonaute RISC catalytic subunit 2
**Agpat9**	231510	1-acylglycerol-3-phosphate O-acyltransferase 9
**Ahdc1**	230793	AT hook, DNA binding motif, containing 1
**Aicda**	11628	activation-induced cytidine deaminase
**Ak4**	11639	adenylate kinase 4
**Akap3**	11642	A kinase (PRKA) anchor protein 3
**Akap6**	238161	A kinase (PRKA) anchor protein 6
**Akr1b10**	67861	aldo-keto reductase family 1, member B10 (aldose reductase)
**Akt2**	11652	thymoma viral proto-oncogene 2
**Alg13**	67574	asparagine-linked glycosylation 13
**Alg8**	381903	asparagine-linked glycosylation 8 (alpha-1,3-glucosyltransferase)
**Alkbh4**	72041	alkB, alkylation repair homolog 4 (E. coli)
**Alkbh6**	233065	alkB, alkylation repair homolog 6 (E. coli)
**Alpk1**	71481	alpha-kinase 1
**Amer1**	72345	APC membrane recruitment 1
**Amn**	93835	amnionless
**Ankrd34a**	545554	ankyrin repeat domain 34A
**Ankrd42**	73845	ankyrin repeat domain 42
**Ano2**	243634	anoctamin 2

**Ap1b1**	11764	adaptor protein complex AP-1, beta 1 subunit
**Ap5z1**	231855	adaptor-related protein complex 5, zeta 1 subunit
**Apeh**	235606	acylpeptide hydrolase
**Apoo**	68316	apolipoprotein O
**Apoo-ps**	621156	apolipoprotein O, pseudogene
**Apoo-ps**	.	.
**Apool**	68117	apolipoprotein O-like
**Aqp2**	11827	aquaporin 2
**Arhgap26**	71302	Rho GTPase activating protein 26
**Arhgdia**	192662	Rho GDP dissociation inhibitor (GDI) alpha
**Arl5c**	217151	ADP-ribosylation factor-like 5C
**Arl6ip5**	65106	ADP-ribosylation factor-like 6 interacting protein 5
**Arl8a**	68724	ADP-ribosylation factor-like 8A
**Arpc5l**	74192	actin related protein 2/3 complex, subunit 5-like
**Arrdc2**	70807	arrestin domain containing 2
**Arsg**	74008	arylsulfatase G
**Asb16**	217217	ankyrin repeat and SOCS box-containing 16
**Asf1b**	66929	anti-silencing function 1B histone chaperone
**Asic1**	11419	acid-sensing (proton-gated) ion channel 1
**Asna1**	56495	arsA arsenite transporter, ATP-binding, homolog 1 (bacterial)
**Asphd2**	72898	aspartate beta-hydroxylase domain containing 2
**Asprv1**	67855	aspartic peptidase, retroviral-like 1
**Aste1**	66595	asteroid homolog 1 (Drosophila)
**Atf5**	107503	activating transcription factor 5
**Atg4a**	666468	autophagy related 4A, cysteine peptidase
**Atp1b1**	11931	ATPase, Na+/K+ transporting, beta 1 polypeptide
**Auts2**	.	.
**Auts2**	319974	autism susceptibility candidate 2
**Avp**	11998	arginine vasopressin
**Awat2**	245532	acyl-CoA wax alcohol acyltransferase 2
**B230217C12Rik**	68127	RIKEN cDNA B230217C12 gene
**B3galt5**	93961	UDP-Gal:betaGlcNAc beta 1,3-galactosyltransferase, polypeptide 5
**B3glct**	381694	beta-3-glucosyltransferase
**B4galnt1**	14421	beta-1,4-N-acetyl-galactosaminyl transferase 1
**B4galnt3**	330406	beta-1,4-N-acetyl-galactosaminyl transferase 3
**B930059L03Rik**	319786	RIKEN cDNA B930059L03 gene
**BC018473**	193217	cDNA sequence BC018473
**BC018473**	.	.
**BC029214**	227622	cDNA sequence BC029214
**BC030867**	217216	cDNA sequence BC030867
**BC039966**	.	.
**BC039966**	407793	cDNA sequence BC039966
**BC048562**	434439	cDNA sequence BC048562

**Bach1**	12013	BTB and CNC homology 1
**Bag3**	29810	BCL2-associated athanogene 3
**Banp**	53325	BTG3 associated nuclear protein
**Barhl1**	54422	BarH-like 1 (Drosophila)
**Baz2a**	116848	bromodomain adjacent to zinc finger domain, 2A
**Bcl6b**	12029	B cell CLL/lymphoma 6, member B
**Best3**	382427	bestrophin 3
**Bet1**	12068	blocked early in transport 1 homolog (S. cerevisiae)
**Bhlha15**	17341	basic helix-loop-helix family, member a15
**Bhmt**	12116	betaine-homocysteine methyltransferase
**Bmpr1b**	12167	bone morphogenetic protein receptor, type 1B
**Boll**	75388	bol, boule-like (Drosophila)
**Brd8**	78656	bromodomain containing 8
**Brinp2**	240843	bone morphogenic protein/retinoic acid inducible neural-specific 2
**Brip1**	237911	BRCA1 interacting protein C-terminal helicase 1
**Btaf1**	107182	BTAF1 RNA polymerase II, B-TFIID transcription factor-associated, (Mot1 homolog, S. cerevisiae)
**Btbd11**	74007	BTB (POZ) domain containing 11
**Btbd18**	100270744	BTB (POZ) domain containing 18
**Btbd19**	.	.
**Btbd19**	78611	BTB (POZ) domain containing 19
**Btbd2**	208198	BTB (POZ) domain containing 2
**Btbd3**	228662	BTB (POZ) domain containing 3
**Bysl**	53414	bystin-like
**C1ql4**	239659	complement component 1, q subcomponent-like 4
**C1rl**	232371	complement component 1, r subcomponent-like
**C330006A16Rik**	.	.
**C330006A16Rik**	109299	RIKEN cDNA C330006A16 gene
**C77080**	97130	expressed sequence C77080
**Cabp1**	29867	calcium binding protein 1
**Cacnb3**	12297	calcium channel, voltage-dependent, beta 3 subunit
**Cacng4**	54377	calcium channel, voltage-dependent, gamma subunit 4
**Cacng5**	140723	calcium channel, voltage-dependent, gamma subunit 5
**Calca**	12310	calcitonin/calcitonin-related polypeptide, alpha
**Calcb**	116903	calcitonin-related polypeptide, beta
**Cald1**	109624	caldesmon 1
**Camk1g**	215303	calcium/calmodulin-dependent protein kinase I gamma
**Cap1**	12331	CAP, adenylate cyclase-associated protein 1 (yeast)
**Car11**	12348	carbonic anhydrase 11
**Car14**	23831	carbonic anhydrase 14
**Car5a**	12352	carbonic anhydrase 5a, mitochondrial
**Cartpt**	27220	CART prepropeptide
**Cbfa2t3**	12398	core-binding factor, runt domain, alpha subunit 2, translocated to, 3 (human)

**Cbln1**	12404	cerebellin 1 precursor protein
**Cbx8**	30951	chromobox 8
**Ccdc113**	244608	coiled-coil domain containing 113
**Ccdc132**	73288	coiled-coil domain containing 132
**Ccdc174**	232236	coiled-coil domain containing 174
**Ccdc24**	381546	coiled-coil domain containing 24
**Ccdc36**	434438	coiled-coil domain containing 36
**Ccdc58**	381045	coiled-coil domain containing 58
**Ccdc60**	269693	coiled-coil domain containing 60
**Ccdc64**	75665	coiled-coil domain containing 64
**Ccdc65**	105833	coiled-coil domain containing 65
**Ccdc74a**	72315	coiled-coil domain containing 74A
**Ccdc85c**	668158	coiled-coil domain containing 85C
**Ccdc93**	70829	coiled-coil domain containing 93
**Ccnd3**	12445	cyclin D3
**Ccng2**	12452	cyclin G2
**Ccnyl1**	227210	cyclin Y-like 1
**Ccr10**	12777	chemokine (C-C motif) receptor 10
**Ccr4**	12773	chemokine (C-C motif) receptor 4
**Ccrl2**	54199	chemokine (C-C motif) receptor-like 2
**Cd163l1**	244233	CD163 molecule-like 1
**Cd164**	53599	CD164 antigen
**Cdc34**	216150	cell division cycle 34
**Cdca3**	14793	cell division cycle associated 3
**Cdca5**	67849	cell division cycle associated 5
**Cdcp2**	242603	CUB domain containing protein 2
**Cdh26**	381409	cadherin-like 26
**Cdh4**	12561	cadherin 4
**Cdk8**	264064	cyclin-dependent kinase 8
**Cdk9**	107951	cyclin-dependent kinase 9 (CDC2-related kinase)
**Cdkn1b**	12576	cyclin-dependent kinase inhibitor 1B
**Cdkn1c**	12577	cyclin-dependent kinase inhibitor 1C (P57)
**Cdt1**	67177	chromatin licensing and DNA replication factor 1
**Cdv3**	321022	carnitine deficiency-associated gene expressed in ventricle 3
**Cecr2**	330409	cat eye syndrome chromosome region, candidate 2
**Cel**	12613	carboxyl ester lipase
**Celrr**	98452	cerebellum expressed regulatory RNA
**Celrr**	.	.
**Cenpi**	102920	centromere protein I
**Cenpl**	70454	centromere protein L
**Cenpu**	71876	centromere protein U
**Cerk**	223753	ceramide kinase
**Ces2h**	436059	carboxylesterase 2H

**Ces3b**	13909	carboxylesterase 3B
**Ces4a**	234677	carboxylesterase 4A
**Chic2**	74277	cysteine-rich hydrophobic domain 2
**Chml**	12663	choroideremia-like
**Chn1**	108699	chimerin 1
**Chrne**	11448	cholinergic receptor, nicotinic, epsilon polypeptide
**Chst1**	76969	carbohydrate (keratan sulfate Gal-6) sulfotransferase 1
**Chst15**	77590	carbohydrate (N-acetylgalactosamine 4-sulfate 6-O) sulfotransferase 15
**Chtop**	66511	chromatin target of PRMT1
**Cisd1**	52637	CDGSH iron sulfur domain 1
**Ckap2l**	70466	cytoskeleton associated protein 2-like
**Cldn11**	18417	claudin 11
**Cldn13**	57255	claudin 13
**Cldn14**	56173	claudin 14
**Cldn15**	60363	claudin 15
**Cldn2**	12738	claudin 2
**Clec16a**	74374	C-type lectin domain family 16, member A
**Clec1b**	56760	C-type lectin domain family 1, member b
**Clec4a1**	269799	C-type lectin domain family 4, member a1
**Clic4**	29876	chloride intracellular channel 4 (mitochondrial)
**Clk2**	12748	CDC-like kinase 2
**Clptm1**	56457	cleft lip and palate associated transmembrane protein 1
**Clstn3**	232370	calsyntenin 3
**Cmas**	12764	cytidine monophospho-N-acetylneuraminic acid synthetase
**Cmtm7**	102545	CKLF-like MARVEL transmembrane domain containing 7
**Cnksr2**	245684	connector enhancer of kinase suppressor of Ras 2
**Cnn2**	12798	calponin 2
**Cnn3**	71994	calponin 3, acidic
**Cntd1**	68107	cyclin N-terminal domain containing 1
**Cog1**	16834	component of oligomeric golgi complex 1
**Cog2**	76332	component of oligomeric golgi complex 2
**Col6a3**	12835	collagen, type VI, alpha 3
**Cox4i1**	12857	cytochrome c oxidase subunit IV isoform 1
**Cox6b1**	110323	cytochrome c oxidase, subunit VIb polypeptide 1
**Cox7a2**	12866	cytochrome c oxidase subunit VIIa 2
**Cox7b**	66142	cytochrome c oxidase subunit VIIb
**Cpeb1**	12877	cytoplasmic polyadenylation element binding protein 1
**Cpt1c**	78070	carnitine palmitoyltransferase 1c
**Crhr1**	12921	corticotropin releasing hormone receptor 1
**Crip1**	12925	cysteine-rich protein 1 (intestinal)
**Csf3**	12985	colony stimulating factor 3 (granulocyte)
**Csrnp3**	77771	cysteine-serine-rich nuclear protein 3
**Cstf1**	67337	cleavage stimulation factor, 3' pre-RNA, subunit 1

**Ctbp2**	13017	C-terminal binding protein 2
**Ctcflos**	74161	CCCTC-binding factor (zinc finger protein)-like, opposite strand
**Ctcflos**	.	.
**Ctdsp2**	52468	CTD (carboxy-terminal domain, RNA polymerase II, polypeptide A) small phosphatase 2
**Cth**	107869	cystathionase (cystathionine gamma-lyase)
**Ctxn2**	381418	cortexin 2
**Cwc25**	67480	CWC25 spliceosome-associated protein homolog (S. cerevisiae)
**Cwc27**	67285	CWC27 spliceosome-associated protein homolog (S. cerevisiae)
**Cxcl10**	15945	chemokine (C-X-C motif) ligand 10
**Cxcr3**	12766	chemokine (C-X-C motif) receptor 3
**Cxxc4**	319478	CXXC finger 4
**Cyb561a3**	225912	cytochrome b561 family, member A3
**Cyth1**	19157	cytohesin 1
**D130040H23Rik**	211135	RIKEN cDNA D130040H23 gene
**D3Bwg0562e**	.	.
**D430020J02Rik**	319545	RIKEN cDNA D430020J02 gene
**D430041D05Rik**	241589	RIKEN cDNA D430041D05 gene
**D630010B17Rik**	328594	RIKEN cDNA D630010B17 gene
**D6Ertd527e**	52372	DNA segment, Chr 6, ERATO Doi 527, expressed
**D7Ertd443e**	71007	DNA segment, Chr 7, ERATO Doi 443, expressed
**Daam2**	76441	dishevelled associated activator of morphogenesis 2
**Dars2**	226539	aspartyl-tRNA synthetase 2 (mitochondrial)
**Dbi**	13167	diazepam binding inhibitor
**Dcaf13**	223499	DDB1 and CUL4 associated factor 13
**Dclre1c**	227525	DNA cross-link repair 1C, PSO2 homolog (S. cerevisiae)
**Dctn2**	69654	dynactin 2
**Ddb2**	107986	damage specific DNA binding protein 2
**Ddx10**	77591	DEAD (Asp-Glu-Ala-Asp) box polypeptide 10
**Ddx17**	67040	DEAD (Asp-Glu-Ala-Asp) box polypeptide 17
**Ddx39b**	53817	DEAD (Asp-Glu-Ala-Asp) box polypeptide 39B
**Ddx5**	13207	DEAD (Asp-Glu-Ala-Asp) box polypeptide 5
**Dennd1b**	329260	DENN/MADD domain containing 1B
**Dgat2l6**	668257	diacylglycerol O-acyltransferase 2-like 6
**Dhx8**	217207	DEAH (Asp-Glu-Ala-His) box polypeptide 8
**Diap2**	54004	diaphanous homolog 2 (Drosophila)
**Dido1**	23856	death inducer-obliterator 1
**Disp2**	214240	dispatched homolog 2 (Drosophila)
**Dlg1**	13383	discs, large homolog 1 (Drosophila)
**Dmtn**	13829	dematin actin binding protein
**Dnajc14**	74330	DnaJ (Hsp40) homolog, subfamily C, member 14
**Dnajc6**	72685	DnaJ (Hsp40) homolog, subfamily C, member 6
**Dnali1**	75563	dynein, axonemal, light intermediate polypeptide 1

**Dner**	227325	delta/notch-like EGF-related receptor
**Dock9**	105445	dedicator of cytokinesis 9
**Dpep3**	71854	dipeptidase 3
**Dpf3**	70127	D4, zinc and double PHD fingers, family 3
**Dppa3**	73708	developmental pluripotency-associated 3
**Drg2**	13495	developmentally regulated GTP binding protein 2
**Drp2**	13497	dystrophin related protein 2
**Dtwd2**	68857	DTW domain containing 2
**Dtx1**	14357	deltex 1 homolog (Drosophila)
**Dusp15**	252864	dual specificity phosphatase-like 15
**Dyrk2**	69181	dual-specificity tyrosine-(Y)-phosphorylation regulated kinase 2
**Dyrk3**	226419	dual-specificity tyrosine-(Y)-phosphorylation regulated kinase 3
**E130006D01Rik**	269683	RIKEN cDNA E130006D01 gene
**E130006D01Rik**	.	.
**E130218I03Rik**	.	.
**E130218I03Rik**	77490	RIKEN cDNA E130218I03 gene
**E2f1**	13555	E2F transcription factor 1
**E2f4**	104394	E2F transcription factor 4
**E2f7**	52679	E2F transcription factor 7
**Ebna1bp2**	69072	EBNA1 binding protein 2
**Ech1**	51798	enoyl coenzyme A hydratase 1, peroxisomal
**Eci3**	69123	enoyl-Coenzyme A delta isomerase 3
**Eda2r**	245527	ectodysplasin A2 receptor
**Edf1**	59022	endothelial differentiation-related factor 1
**Efna1**	13636	ephrin A1
**Efna2**	13637	ephrin A2
**Egr4**	13656	early growth response 4
**Eid3**	66341	EP300 interacting inhibitor of differentiation 3
**Eif4b**	75705	eukaryotic translation initiation factor 4B
**Eif4e3**	66892	eukaryotic translation initiation factor 4E member 3
**Elavl2**	15569	ELAV (embryonic lethal, abnormal vision, Drosophila)-like 2 (Hu antigen B)
**Elavl4**	15572	ELAV (embryonic lethal, abnormal vision, Drosophila)-like 4 (Hu antigen D)
**Emb**	13723	embigin
**Eme1**	268465	essential meiotic endonuclease 1 homolog 1 (S. pombe)
**Endou**	19011	endonuclease, polyU-specific
**Eno4**	226265	enolase 4
**Enpep**	13809	glutamyl aminopeptidase
**Entpd6**	12497	ectonucleoside triphosphate diphosphohydrolase 6
**Epb4.1**	269587	erythrocyte protein band 4.1
**Epc1**	13831	enhancer of polycomb homolog 1 (Drosophila)
**Ephb1**	270190	Eph receptor B1
**Ephb2**	13844	Eph receptor B2

**Eppin**	75526	epididymal peptidase inhibitor
**Ercc3**	13872	excision repair cross-complementing rodent repair deficiency, complementation group 3
**Erg**	13876	avian erythroblastosis virus E-26 (v-ets) oncogene related
**Ergic2**	67456	ERGIC and golgi 2
**Erh**	13877	enhancer of rudimentary homolog (Drosophila)
**Esrp2**	77411	epithelial splicing regulatory protein 2
**Esrra**	26379	estrogen related receptor, alpha
**Eva1c**	70967	eva-1 homolog C (C. elegans)
**Evi5l**	213027	ecotropic viral integration site 5 like
**Exoc3l**	277978	exocyst complex component 3-like
**Ext1**	14042	exostoses (multiple) 1
**Ezh1**	14055	enhancer of zeste homolog 1 (Drosophila)
**F3**	14066	coagulation factor III
**F5**	14067	coagulation factor V
**Fabp3**	14077	fatty acid binding protein 3, muscle and heart
**Faim2**	72393	Fas apoptotic inhibitory molecule 2
**Fam109a**	231717	family with sequence similarity 109, member A
**Fam162a**	70186	family with sequence similarity 162, member A
**Fam169a**	320557	family with sequence similarity 169, member A
**Fam171a1**	269233	family with sequence similarity 171, member A1
**Fam19a5**	106014	family with sequence similarity 19, member A5
**Fam210a**	108654	family with sequence similarity 210, member A
**Fam222a**	433940	family with sequence similarity 222, member A
**Fam229a**	68233	family with sequence similarity 229, member A
**Fam78b**	226610	family with sequence similarity 78, member B
**Fam83d**	71878	family with sequence similarity 83, member D
**Fancl**	67030	Fanconi anemia, complementation group L
**Farp1**	223254	FERM, RhoGEF (Arhgef) and pleckstrin domain protein 1 (chondrocyte-derived)
**Fastkd5**	380601	FAST kinase domains 5
**Fbln5**	23876	fibulin 5
**Fbrsl1**	381668	fibrosin-like 1
**Fbxl19**	233902	F-box and leucine-rich repeat protein 19
**Fbxl5**	242960	F-box and leucine-rich repeat protein 5
**Fcgr4**	246256	Fc receptor, IgG, low affinity IV
**Fchsd1**	319262	FCH and double SH3 domains 1
**Fen1**	14156	flap structure specific endonuclease 1
**Fes**	14159	feline sarcoma oncogene
**Fgf14**	14169	fibroblast growth factor 14
**Fgf23**	64654	fibroblast growth factor 23
**Fgf3**	14174	fibroblast growth factor 3
**Fhod1**	234686	formin homology 2 domain containing 1
**Fignl2**	668225	fidgetin-like 2

**Fis1**	66437	fission 1 (mitochondrial outer membrane) homolog (yeast)
**Fjx1**	14221	four jointed box 1 (Drosophila)
**Fmnl1**	57778	formin-like 1
**Fndc5**	384061	fibronectin type III domain containing 5
**Foxd2**	17301	forkhead box D2
**Foxg1**	15228	forkhead box G1
**Foxp1**	108655	forkhead box P1
**Foxs1**	14239	forkhead box S1
**Fpgt**	75540	fucose-1-phosphate guanylyltransferase
**Frmd4b**	232288	FERM domain containing 4B
**Frmd6**	319710	FERM domain containing 6
**Frmd8**	67457	FERM domain containing 8
**Frmpd3**	245643	FERM and PDZ domain containing 3
**Fscn1**	14086	fascin homolog 1, actin bundling protein (Strongylocentrotus purpuratus)
**Fzd7**	14369	frizzled homolog 7 (Drosophila)
**Gabrd**	14403	gamma-aminobutyric acid (GABA) A receptor, subunit delta
**Gal3st3**	545276	galactose-3-O-sulfotransferase 3
**Ganc**	76051	glucosidase, alpha; neutral C
**Gcnt4**	218476	glucosaminyl (N-acetyl) transferase 4, core 2 (beta-1,6-N-acetylglucosaminyltransferase)
**Gdap1l1**	228858	ganglioside-induced differentiation-associated protein 1-like 1
**Gdf11**	14561	growth differentiation factor 11
**Ghrh**	14601	growth hormone releasing hormone
**Gid8**	76425	GID complex subunit 8 homolog (S. cerevisiae)
**Gin1**	252876	gypsy retrotransposon integrase 1
**Gip**	14607	gastric inhibitory polypeptide
**Gja8**	14616	gap junction protein, alpha 8
**Gjb5**	14622	gap junction protein, beta 5
**Glra4**	14657	glycine receptor, alpha 4 subunit
**Gltp**	56356	glycolipid transfer protein
**Gm10584**	100043682	predicted gene 10584
**Gm11562**	100502803	predicted gene 11562
**Gm11595**	100040276	predicted gene 11595
**Gm12695**	620779	predicted gene 12695
**Gm12718**	622167	predicted gene 12718
**Gm12718**	.	.
**Gm13238**	.	.
**Gm13238**	236069	carnitine deficiency-associated gene expressed in ventricle 3 pseudogene
**Gm13826**	.	.
**Gm13889**	620695	predicted gene 13889
**Gm14023**	.	.
**Gm14023**	100503468	predicted gene 14023
**Gm14164**	.	.

**Gm14164**	791417	predicted gene 14164
**Gm15412**	.	.
**Gm15412**	670727	predicted gene 15412
**Gm1564**	268491	predicted gene 1564
**Gm15663**	.	.
**Gm15663**	327812	predicted gene 15663
**Gm15760**	.	.
**Gm15760**	100042948	mitochondrial ribosomal protein S18B pseudogene
**Gm16063**	330183	predicted gene 16063
**Gm16063**	.	.
**Gm166**	233899	predicted gene 166
**Gm16701**	.	.
**Gm16701**	.	.
**Gm16701**	100503962	predicted gene, 16701
**Gm17296**	212728	predicted gene, 17296
**Gm2061**	100039138	predicted gene 2061
**Gm2061**	.	.
**Gm2109**	.	.
**Gm2109**	100039222	predicted gene 2109
**Gm2694**	.	.
**Gm2694**	100040294	predicted gene 2694
**Gm4013**	100042757	predicted gene 4013
**Gm4262**	.	.
**Gm4262**	100043147	predicted gene 4262
**Gm4980**	100503386	predicted gene 4980
**Gm5166**	.	.
**Gm5166**	382233	DMRT-like family pseudogene
**Gm53**	193022	predicted gene 53
**Gm53**	.	.
**Gm5475**	.	.
**Gm5475**	432982	predicted gene 5475
**Gm5477**	432986	type II keratin Kb16P
**Gm6042**	574415	type II keratin Kb32P
**Gm614**	245536	predicted gene 614
**Gm7157**	.	.
**Gm7538**	665194	predicted gene 7538
**Gm826**	329554	predicted gene 826
**Gmfg**	63986	glia maturation factor, gamma
**Gnai3**	14679	guanine nucleotide binding protein (G protein), alpha inhibiting 3
**Gnas**	14683	GNAS (guanine nucleotide binding protein, alpha stimulating) complex locus
**Gnb3**	14695	guanine nucleotide binding protein (G protein), beta 3
**Gopc**	94221	golgi associated PDZ and coiled-coil motif containing

**Gpc1**	14733	glypican 1
**Gpc6**	23888	glypican 6
**Gpr133**	.	.
**Gpr143**	18241	G protein-coupled receptor 143
**Gpr182**	11536	G protein-coupled receptor 182
**Gpr27**	14761	G protein-coupled receptor 27
**Gpr3**	14748	G-protein coupled receptor 3
**Gpr84**	80910	G protein-coupled receptor 84
**Gprc5b**	64297	G protein-coupled receptor, family C, group 5, member B
**Gpsm1**	67839	G-protein signalling modulator 1 (AGS3-like, C. elegans)
**Gpx7**	67305	glutathione peroxidase 7
**Grik3**	14807	glutamate receptor, ionotropic, kainate 3
**Grip1**	74053	glutamate receptor interacting protein 1
**Grpr**	14829	gastrin releasing peptide receptor
**Gsdma**	57911	gasdermin A
**Gsg1l**	269994	GSG1-like
**Gsr**	14782	glutathione reductase
**Gtf2a2**	235459	general transcription factor II A, 2
**Gtf3c6**	67371	general transcription factor IIIC, polypeptide 6, alpha
**Gtsf1**	74174	gametocyte specific factor 1
**Hamp2**	66438	hepcidin antimicrobial peptide 2
**Haus5**	71909	HAUS augmin-like complex, subunit 5
**Hcn3**	15168	hyperpolarization-activated, cyclic nucleotide-gated K+ 3
**Hdac8**	70315	histone deacetylase 8
**Helz**	78455	helicase with zinc finger domain
**Hepacam2**	101202	HEPACAM family member 2
**Hexim1**	192231	hexamethylene bis-acetamide inducible 1
**Higd1c**	380975	HIG1 domain family, member 1C
**Higd2a**	67044	HIG1 domain family, member 2A
**Hist2h2aa1**	15267	histone cluster 2, H2aa1
**Hist2h2aa2**	319192	histone cluster 2, H2aa2
**Hist2h3c1**	15077	histone cluster 2, H3c1
**Hist2h3c2**	97114	histone cluster 2, H3c2
**Hlf**	217082	hepatic leukemia factor
**Hmx2**	15372	H6 homeobox 2
**Hnrnpl**	15388	heterogeneous nuclear ribonucleoprotein L
**Hook1**	77963	hook homolog 1 (Drosophila)
**Hook2**	170833	hook homolog 2 (Drosophila)
**Hoxa5**	15402	homeobox A5
**Hoxa6**	15403	homeobox A6
**Hoxb2**	103889	homeobox B2
**Hoxc8**	15426	homeobox C8
**Hp**	15439	haptoglobin

**Hpdl**	242642	4-hydroxyphenylpyruvate dioxygenase-like
**Hrh3**	99296	histamine receptor H3
**Hs6st3**	50787	heparan sulfate 6-O-sulfotransferase 3
**Hsd17b14**	66065	hydroxysteroid (17-beta) dehydrogenase 14
**Hsd17b2**	15486	hydroxysteroid (17-beta) dehydrogenase 2
**Hunk**	26559	hormonally upregulated Neu-associated kinase
**Ier2**	15936	immediate early response 2
**Iffo2**	212632	intermediate filament family orphan 2
**Ifitm10**	320802	interferon induced transmembrane protein 10
**Ift122**	81896	intraflagellar transport 122
**Ift46**	76568	intraflagellar transport 46
**Igbp1**	18518	immunoglobulin (CD79A) binding protein 1
**Igbp1b**	50540	immunoglobulin (CD79A) binding protein 1b
**Igf2bp1**	140486	insulin-like growth factor 2 mRNA binding protein 1
**Igfals**	16005	insulin-like growth factor binding protein, acid labile subunit
**Igsf9**	93842	immunoglobulin superfamily, member 9
**Il12b**	16160	interleukin 12b
**Il4ra**	16190	interleukin 4 receptor, alpha
**Ints5**	109077	integrator complex subunit 5
**Ipmk**	69718	inositol polyphosphate multikinase
**Iqsec1**	232227	IQ motif and Sec7 domain 1
**Iqsec2**	245666	IQ motif and Sec7 domain 2
**Irf9**	16391	interferon regulatory factor 9
**Irgm1**	15944	immunity-related GTPase family M member 1
**Irs4**	16370	insulin receptor substrate 4
**Isyna1**	71780	myo-inositol 1-phosphate synthase A1
**Itga3**	16400	integrin alpha 3
**Itgb7**	16421	integrin beta 7
**Itpripl2**	319622	inositol 1,4,5-triphosphate receptor interacting protein-like 2
**Junb**	16477	jun B proto-oncogene
**Kcnab2**	16498	potassium voltage-gated channel, shaker-related subfamily, beta member 2
**Kcnc4**	99738	potassium voltage gated channel, Shaw-related subfamily, member 4
**Kcnd3**	56543	potassium voltage-gated channel, Shal-related family, member 3
**Kcnd3os**	.	.
**Kcnd3os**	78635	potassium voltage-gated channel, Shal-related family, member 3, opposite strand
**Kcne2**	246133	potassium voltage-gated channel, Isk-related subfamily, gene 2
**Kcnj10**	16513	potassium inwardly-rectifying channel, subfamily J, member 10
**Kcnj12**	16515	potassium inwardly-rectifying channel, subfamily J, member 12
**Kcnk15**	241769	potassium channel, subfamily K, member 15
**Kctd12**	239217	potassium channel tetramerisation domain containing 12
**Kctd13**	233877	potassium channel tetramerisation domain containing 13
**Kctd3**	226823	potassium channel tetramerisation domain containing 3

**Khsrp**	16549	KH-type splicing regulatory protein
**Kif20a**	19348	kinesin family member 20A
**Kif22**	110033	kinesin family member 22
**Kif5a**	16572	kinesin family member 5A
**Kifc3**	16582	kinesin family member C3
**Kiss1**	280287	KiSS-1 metastasis-suppressor
**Klf15**	66277	Kruppel-like factor 15
**Klhdc8a**	213417	kelch domain containing 8A
**Klhl36**	234796	kelch-like 36
**Klhl42**	232539	kelch-like 42
**Klk1b4**	18048	kallikrein 1-related pepidase b4
**Klk1b5**	16622	kallikrein 1-related peptidase b5
**Klk8**	259277	kallikrein related-peptidase 8
**Klrb1c**	17059	killer cell lectin-like receptor subfamily B member 1C
**Kpna7**	381686	karyopherin alpha 7 (importin alpha 8)
**Krt10**	16661	keratin 10
**Krt13**	16663	keratin 13
**Krt2**	16681	keratin 2
**Krt5**	110308	keratin 5
**Krt6a**	16687	keratin 6A
**Krt77**	406220	keratin 77
**Krtap17-1**	77914	keratin associated protein 17-1
**Krtap2-4**	71453	keratin associated protein 2-4
**Krtap4-6**	68768	keratin associated protein 4-6
**Krtap4-8**	665992	keratin associated protein 4-8
**Krtap4-9**	665998	keratin associated protein 4-9
**Krtap9-3**	75586	keratin associated protein 9-3
**Krtcap2**	66059	keratinocyte associated protein 2
**LOC100503676**	.	.
**LOC100504703**	.	.
**LOC381967**	.	.
**Lama5**	16776	laminin, alpha 5
**Lce1b**	68720	late cornified envelope 1B
**Lck**	16818	lymphocyte protein tyrosine kinase
**Ldhb**	16832	lactate dehydrogenase B
**Ldlrap1**	100017	low density lipoprotein receptor adaptor protein 1
**Ldoc1l**	223732	leucine zipper, down-regulated in cancer 1-like
**Lef1**	16842	lymphoid enhancer binding factor 1
**Leo1**	235497	Leo1, Paf1/RNA polymerase II complex component, homolog (S. cerevisiae)
**Lepr**	16847	leptin receptor
**Lin28a**	83557	lin-28 homolog A (C. elegans)
**Lingo4**	320747	leucine rich repeat and Ig domain containing 4

**Liph**	239759	lipase, member H
**Litaf**	56722	LPS-induced TN factor
**Lix1l**	280411	Lix1-like
**Lkaaear1**	277496	LKAAEAR motif containing 1 (IKAAEAR murine motif)
**Llgl1**	16897	lethal giant larvae homolog 1 (Drosophila)
**Lmo4**	16911	LIM domain only 4
**Lmod1**	93689	leiomodin 1 (smooth muscle)
**Lmtk2**	231876	lemur tyrosine kinase 2
**Lpar5**	381810	lysophosphatidic acid receptor 5
**Lpin2**	64898	lipin 2
**Lpin3**	64899	lipin 3
**Lrig2**	269473	leucine-rich repeats and immunoglobulin-like domains 2
**Lrp1**	16971	low density lipoprotein receptor-related protein 1
**Lrp8**	16975	low density lipoprotein receptor-related protein 8, apolipoprotein e receptor
**Lrrc38**	242735	leucine rich repeat containing 38
**Lrrc4c**	241568	leucine rich repeat containing 4C
**Lrriq3**	74435	leucine-rich repeats and IQ motif containing 3
**Lrrn1**	16979	leucine rich repeat protein 1, neuronal
**Lrwd1**	71735	leucine-rich repeats and WD repeat domain containing 1
**Luzp4**	105246601	leucine zipper protein 4
**Luzp4**	434865	leucine zipper protein 4
**Ly6g5c**	114652	lymphocyte antigen 6 complex, locus G5C
**Lyzl6**	69444	lysozyme-like 6
**Macf1**	11426	microtubule-actin crosslinking factor 1
**Maf**	17132	avian musculoaponeurotic fibrosarcoma (v-maf) AS42 oncogene homolog
**Maged1**	94275	melanoma antigen, family D, 1
**Magi3**	99470	membrane associated guanylate kinase, WW and PDZ domain containing 3
**Magt1**	67075	magnesium transporter 1
**Malat1**	.	.
**Malat1**	72289	metastasis associated lung adenocarcinoma transcript 1 (non-coding RNA)
**Maneal**	215090	mannosidase, endo-alpha-like
**Mansc4**	545893	MANSC domain containing 4
**Map2k6**	26399	mitogen-activated protein kinase kinase 6
**Map3k1**	26401	mitogen-activated protein kinase kinase kinase 1
**Map6**	17760	microtubule-associated protein 6
**Mapkapk3**	102626	mitogen-activated protein kinase-activated protein kinase 3
**Marcks**	17118	myristoylated alanine rich protein kinase C substrate
**Marveld3**	73608	MARVEL (membrane-associating) domain containing 3
**Mbd4**	17193	methyl-CpG binding domain protein 4
**Mc3r**	17201	melanocortin 3 receptor
**Mcm2**	17216	minichromosome maintenance deficient 2 mitotin (S. cerevisiae)
**Mdk**	17242	midkine
**Mdm1**	17245	transformed mouse 3T3 cell double minute 1

**Me1**	17436	malic enzyme 1, NADP(+)-dependent, cytosolic
**Med18**	67219	mediator complex subunit 18
**Med19**	381379	mediator complex subunit 19
**Med20**	56771	mediator complex subunit 20
**Meig1**	104362	meiosis expressed gene 1
**Mettl11b**	240879	methyltransferase like 11B
**Mfsd5**	106073	major facilitator superfamily domain containing 5
**Mgat3**	17309	mannoside acetylglucosaminyltransferase 3
**Mid1**	17318	midline 1
**Mid2**	23947	midline 2
**Mif4gd**	69674	MIF4G domain containing
**Mir130a**	.	.
**Mir130a**	387149	microRNA 130a
**Mir138-1**	387156	microRNA 138-1
**Mir138-1**	.	.
**Mir141**	.	.
**Mir141**	387159	microRNA 141
**Mir17**	723905	microRNA 17
**Mir17**	.	.
**Mir17hg**	.	.
**Mir17hg**	75957	Mir17 host gene (non-protein coding)
**Mir18**	.	.
**Mir18**	387135	microRNA 18
**Mir181c**	.	.
**Mir181c**	723819	microRNA 181c
**Mir181d**	100049549	microRNA 181d
**Mir181d**	.	.
**Mir184**	.	.
**Mir184**	387179	microRNA 184
**Mir1893**	100316773	microRNA 1893
**Mir1893**	.	.
**Mir1895**	100316832	microRNA 1895
**Mir1895**	.	.
**Mir1905**	100316808	microRNA 1905
**Mir1905**	.	.
**Mir1934**	.	.
**Mir1934**	100316840	microRNA 1934
**Mir1940**	.	.
**Mir195a**	.	.
**Mir195a**	387190	microRNA 195a
**Mir1966**	100316712	microRNA 1966
**Mir1966**	.	.
**Mir1981**	.	.

**Mir1981**	100316821	microRNA 1981
**Mir19a**	.	.
**Mir19a**	723891	microRNA 19a
**Mir19b-1**	.	.
**Mir19b-1**	751527	microRNA 19b-1
**Mir200c**	.	.
**Mir200c**	723944	microRNA 200c
**Mir20a**	.	.
**Mir20a**	387139	microRNA 20a
**Mir22**	.	.
**Mir22**	387141	microRNA 22
**Mir22hg**	.	.
**Mir22hg**	100042498	Mir22 host gene (non-protein coding)
**Mir26a-2**	723962	microRNA 26a-2
**Mir26a-2**	.	.
**Mir2861**	.	.
**Mir2861**	100499514	microRNA 2861
**Mir3065**	100526518	microRNA 3065
**Mir3076**	.	.
**Mir3076**	100526475	microRNA 3076
**Mir3088**	.	.
**Mir3088**	100526498	microRNA 3088
**Mir3093**	.	.
**Mir3093**	100526540	microRNA 3093
**Mir30c-1**	.	.
**Mir30c-1**	387227	microRNA 30c-1
**Mir30f**	102466650	microRNA 30f
**Mir3102**	.	.
**Mir3102**	100526508	microRNA 3102
**Mir3108**	.	.
**Mir3108**	100526511	microRNA 3108
**Mir326**	723840	microRNA 326
**Mir326**	.	.
**Mir3475**	.	.
**Mir3475**	100499512	microRNA 3475
**Mir3960**	100628606	microRNA 3960
**Mir449a**	.	.
**Mir449a**	723868	microRNA 449a
**Mir449b**	.	.
**Mir449b**	100190765	microRNA 449b
**Mir497**	751537	microRNA 497
**Mir497b**	102465891	microRNA 497b
**Mir497b**	.	.

**Mir499**	735275	microRNA 499
**Mir499**	.	.
**Mir5114**	100628619	microRNA 5114
**Mir5114**	.	.
**Mir5126**	.	.
**Mir5126**	100628595	microRNA 5126
**Mir5130**	.	.
**Mir5130**	100628599	microRNA 5130
**Mir5136**	100628605	microRNA 5136
**Mir5136**	.	.
**Mir6391**	.	.
**Mir6391**	102465208	microRNA 6391
**Mir672**	.	.
**Mir672**	751535	microRNA 672
**Mir676**	.	.
**Mir676**	751534	microRNA 676
**Mir6916**	.	.
**Mir6916**	102465552	microRNA 6916
**Mir6931**	102466988	microRNA 6931
**Mir6931**	.	.
**Mir6934**	.	.
**Mir6934**	102465563	microRNA 6934
**Mir6959**	.	.
**Mir6959**	102465981	microRNA 6959
**Mir6975**	.	.
**Mir6975**	102465589	microRNA 6975
**Mir6977**	102465590	microRNA 6977
**Mir6977**	.	.
**Mir7003**	.	.
**Mir7003**	102465607	microRNA 7003
**Mir7004**	102466784	microRNA 7004
**Mir702**	735283	microRNA 702
**Mir702**	.	.
**Mir7022**	.	.
**Mir7022**	102465619	microRNA 7022
**Mir7054**	.	.
**Mir7054**	102466218	microRNA 7054
**Mir7072**	102466800	microRNA 7072
**Mir7072**	.	.
**Mir7088**	102466914	microRNA 7088
**Mir7088**	.	.
**Mir7222**	.	.
**Mir7222**	102465704	microRNA 7222

**Mir7233**	102466996	microRNA 7233
**Mir7235**	102466822	microRNA 7235
**Mir7235**	.	.
**Mir763**	791076	microRNA 763
**Mir763**	.	.
**Mir7678**	.	.
**Mir7678**	102465780	microRNA 7678
**Mir7686**	102465788	microRNA mir-7686
**Mir7687**	.	.
**Mir7687**	102466849	microRNA 7687
**Mir802**	.	.
**Mir802**	791074	microRNA 802
**Mir8103**	102465893	microRNA 8103
**Mir8103**	.	.
**Mir8107**	.	.
**Mir8107**	102466885	microRNA 8107
**Mir9-1**	387133	microRNA 9-1
**Mir92-1**	.	.
**Mir92-1**	751549	microRNA 92-1
**Mirlet7k**	.	.
**Mixl1**	27217	Mix1 homeobox-like 1 (Xenopus laevis)
**Mlxipl**	58805	MLX interacting protein-like
**Mmd**	67468	monocyte to macrophage differentiation-associated
**Mmp23**	26561	matrix metallopeptidase 23
**Mmp24**	17391	matrix metallopeptidase 24
**Mn1**	433938	meningioma 1
**Mnat1**	17420	menage a trois 1
**Mon1b**	270096	MON1 homolog b (yeast)
**Mon2**	67074	MON2 homolog (yeast)
**Mospd3**	68929	motile sperm domain containing 3
**Mpdu1**	24070	mannose-P-dolichol utilization defect 1
**Mpped2**	77015	metallophosphoesterase domain containing 2
**Mpv17**	17527	MpV17 mitochondrial inner membrane protein
**Mrc2**	17534	mannose receptor, C type 2
**Mrgpre**	244238	MAS-related GPR, member E
**Mroh1**	223658	maestro heat-like repeat family member 1
**Mrpl18**	67681	mitochondrial ribosomal protein L18
**Mrpl27**	94064	mitochondrial ribosomal protein L27
**Mrpl32**	75398	mitochondrial ribosomal protein L32
**Mrps17**	66258	mitochondrial ribosomal protein S17
**Mrps6**	121022	mitochondrial ribosomal protein S6
**Mrvi1**	17540	MRV integration site 1
**Msi1**	17690	musashi RNA-binding protein 1

**Msmp**	100039672	microseminoprotein, prostate associated
**Msrb1**	27361	methionine sulfoxide reductase B1
**Msrb3**	320183	methionine sulfoxide reductase B3
**Mst1**	15235	macrophage stimulating 1 (hepatocyte growth factor-like)
**Mt4**	17752	metallothionein 4
**Mterfd3**	.	.
**Mtmr4**	170749	myotubularin related protein 4
**Muc6**	353328	mucin 6, gastric
**Muc6**	.	.
**Mvd**	192156	mevalonate (diphospho) decarboxylase
**Mvp**	78388	major vault protein
**Mxd4**	17122	Max dimerization protein 4
**Myadm**	50918	myeloid-associated differentiation marker
**Myadml2**	68515	myeloid-associated differentiation marker-like 2
**Mybph**	53311	myosin binding protein H
**Myl10**	59310	myosin, light chain 10, regulatory
**Myo1a**	432516	myosin IA
**Myo9a**	270163	myosin IXa
**Myog**	17928	myogenin
**Naa35**	78689	N(alpha)-acetyltransferase 35, NatC auxiliary subunit
**Nalcn**	338370	sodium leak channel, non-selective
**Ncapg2**	76044	non-SMC condensin II complex, subunit G2
**Nckap5**	210356	NCK-associated protein 5
**Nctc1**	330677	non-coding transcript 1
**Nctc1**	.	.
**Ndnl2**	66647	necdin-like 2
**Ndufa3**	66091	NADH dehydrogenase (ubiquinone) 1 alpha subcomplex, 3
**Ndufa9**	66108	NADH dehydrogenase (ubiquinone) 1 alpha subcomplex, 9
**Ndufb2**	68198	NADH dehydrogenase (ubiquinone) 1 beta subcomplex, 2
**Negr1**	320840	neuronal growth regulator 1
**Neurod4**	11923	neurogenic differentiation 4
**Nfib**	18028	nuclear factor I/B
**Ngdn**	68966	neuroguidin, EIF4E binding protein
**Ngf**	18049	nerve growth factor
**Nhsl2**	100042480	NHS-like 2
**Nipal3**	74552	NIPA-like domain containing 3
**Nisch**	64652	nischarin
**Nkd1**	93960	naked cuticle 1 homolog (Drosophila)
**Nlrp6**	101613	NLR family, pyrin domain containing 6
**Nme5**	75533	NME/NM23 family member 5
**Nme9**	.	.
**Nme9**	623534	NME/NM23 family member 9
**Nmnat2**	226518	nicotinamide nucleotide adenylyltransferase 2

**Nmur1**	14767	neuromedin U receptor 1
**Nnat**	18111	neuronatin
**Nop16**	28126	NOP16 nucleolar protein
**Noxa1**	241275	NADPH oxidase activator 1
**Nphp4**	260305	nephronophthisis 4 (juvenile) homolog (human)
**Npl**	74091	N-acetylneuraminate pyruvate lyase
**Nppa**	230899	natriuretic peptide type A
**Nps**	100043254	neuropeptide S
**Nptxr**	73340	neuronal pentraxin receptor
**Nr1h3**	22259	nuclear receptor subfamily 1, group H, member 3
**Nr4a1**	15370	nuclear receptor subfamily 4, group A, member 1
**Nr5a2**	26424	nuclear receptor subfamily 5, group A, member 2
**Nt5c1a**	230718	5'-nucleotidase, cytosolic IA
**Nt5e**	23959	5' nucleotidase, ecto
**Ntm**	235106	neurotrimin
**Nuak2**	74137	NUAK family, SNF1-like kinase, 2
**Nyap1**	243300	neuronal tyrosine-phosphorylated phosphoinositide 3-kinase adaptor 1
**Oas1f**	243262	2'-5' oligoadenylate synthetase 1F
**Oasl1**	231655	2'-5' oligoadenylate synthetase-like 1
**Ocln**	18260	occludin
**Ogg1**	18294	8-oxoguanine DNA-glycosylase 1
**Olfm3**	229759	olfactomedin 3
**Olfr1410**	258484	olfactory receptor 1410
**Olfr1412**	258274	olfactory receptor 1412
**Olfr1414**	259041	olfactory receptor 1414
**Olfr1416**	259040	olfactory receptor 1416
**Olfr214**	258754	olfactory receptor 214
**Olfr283**	259038	olfactory receptor 283
**Olfr286**	629524	olfactory receptor 286
**Olfr287**	634104	olfactory receptor 287
**Olfr522**	258954	olfactory receptor 522
**Olfr62**	18363	olfactory receptor 62
**Olfr632**	259123	olfactory receptor 632
**Opa3**	403187	optic atrophy 3
**Opn3**	13603	opsin 3
**Ormdl2**	66844	ORM1-like 2 (S. cerevisiae)
**Oscar**	232790	osteoclast associated receptor
**Oser1**	66680	oxidative stress responsive serine rich 1
**Otub2**	68149	OTU domain, ubiquitin aldehyde binding 2
**P2rx7**	18439	purinergic receptor P2X, ligand-gated ion channel, 7
**P2ry2**	18442	purinergic receptor P2Y, G-protein coupled 2
**Pacsin3**	80708	protein kinase C and casein kinase substrate in neurons 3
**Pafah2**	100163	platelet-activating factor acetylhydrolase 2

**Pak6**	214230	p21 protein (Cdc42/Rac)-activated kinase 6
**Palmd**	114301	palmdelphin
**Papolb**	56522	poly (A) polymerase beta (testis specific)
**Pappa2**	23850	pappalysin 2
**Paqr9**	75552	progestin and adipoQ receptor family member IX
**Pard3**	93742	par-3 family cell polarity regulator
**Pars2**	230577	prolyl-tRNA synthetase (mitochondrial)(putative)
**Pawr**	114774	PRKC, apoptosis, WT1, regulator
**Pax6**	18508	paired box 6
**Pax6os1**	402728	paired box 6 opposite strand 1
**Pax6os1**	.	.
**Paxip1**	55982	PAX interacting (with transcription-activation domain) protein 1
**Pcdh9**	211712	protocadherin 9
**Pcdhga5**	93713	protocadherin gamma subfamily A, 5
**Pcp4**	18546	Purkinje cell protein 4
**Pcyt1b**	236899	phosphate cytidylyltransferase 1, choline, beta isoform
**Pdcl**	67466	phosducin-like
**Pddc1**	213350	Parkinson disease 7 domain containing 1
**Pde10a**	23984	phosphodiesterase 10A
**Pde1b**	18574	phosphodiesterase 1B, Ca2+-calmodulin dependent
**Pde3a**	54611	phosphodiesterase 3A, cGMP inhibited
**Pde6h**	78600	phosphodiesterase 6H, cGMP-specific, cone, gamma
**Pdia4**	12304	protein disulfide isomerase associated 4
**Pdyn**	18610	prodynorphin
**Pes1**	64934	pescadillo homolog 1, containing BRCT domain (zebrafish)
**Pfdn4**	109054	prefoldin 4
**Pfdn6**	14976	prefoldin subunit 6
**Pgbd5**	209966	piggyBac transposable element derived 5
**Pgk1**	18655	phosphoglycerate kinase 1
**Phactr3**	74189	phosphatase and actin regulator 3
**Phb**	18673	prohibitin
**Phf21a**	192285	PHD finger protein 21A
**Phf5a**	68479	PHD finger protein 5A
**Phip**	83946	pleckstrin homology domain interacting protein
**Pianp**	319352	PILR alpha associated neural protein
**Pid1**	98496	phosphotyrosine interaction domain containing 1
**Pik3cd**	18707	phosphatidylinositol 3-kinase catalytic delta polypeptide
**Pin4**	69713	protein (peptidyl-prolyl cis/trans isomerase) NIMA-interacting, 4 (parvulin)
**Pitpnm2**	19679	phosphatidylinositol transfer protein, membrane-associated 2
**Piwil1**	57749	piwi-like RNA-mediated gene silencing 1
**Pklr**	18770	pyruvate kinase liver and red blood cell
**Pla2g2a**	18780	phospholipase A2, group IIA (platelets, synovial fluid)
**Pla2g2a**	.	.

**Pla2g2d**	18782	phospholipase A2, group IID
**Plcxd1**	403178	phosphatidylinositol-specific phospholipase C, X domain containing 1
**Plk2**	20620	polo-like kinase 2
**Pls1**	102502	plastin 1 (I-isoform)
**Plxna1**	18844	plexin A1
**Pmvk**	68603	phosphomevalonate kinase
**Polg**	18975	polymerase (DNA directed), gamma
**Polh**	80905	polymerase (DNA directed), eta (RAD 30 related)
**Polr3gl**	69870	polymerase (RNA) III (DNA directed) polypeptide G like
**Pou3f1**	18991	POU domain, class 3, transcription factor 1
**Pou6f1**	19009	POU domain, class 6, transcription factor 1
**Pparg**	19016	peroxisome proliferator activated receptor gamma
**Ppfia4**	68507	protein tyrosine phosphatase, receptor type, f polypeptide (PTPRF), interacting protein (liprin), alpha 4
**Ppfibp1**	67533	PTPRF interacting protein, binding protein 1 (liprin beta 1)
**Ppif**	105675	peptidylprolyl isomerase F (cyclophilin F)
**Ppip5k2**	227399	diphosphoinositol pentakisphosphate kinase 2
**Ppp1r14d**	72112	protein phosphatase 1, regulatory (inhibitor) subunit 14D
**Ppp4r1l-ps**	.	.
**Ppp4r1l-ps**	100043911	protein phosphatase 4, regulatory subunit 1-like, pseudogene
**Ppp4r4**	74521	protein phosphatase 4, regulatory subunit 4
**Pptc7**	320717	PTC7 protein phosphatase homolog (S. cerevisiae)
**Ppy**	19064	pancreatic polypeptide
**Prex1**	277360	phosphatidylinositol-3,4,5-trisphosphate-dependent Rac exchange factor 1
**Prickle2**	243548	prickle homolog 2 (Drosophila)
**Prkcb**	18751	protein kinase C, beta
**Prkce**	18754	protein kinase C, epsilon
**Prmt8**	381813	protein arginine N-methyltransferase 8
**Prok1**	246691	prokineticin 1
**Prss36**	77613	protease, serine 36
**Psma2**	19166	proteasome (prosome, macropain) subunit, alpha type 2
**Psmb6**	19175	proteasome (prosome, macropain) subunit, beta type 6
**Psmd10**	53380	proteasome (prosome, macropain) 26S subunit, non-ATPase, 10
**Ptbp1**	19205	polypyrimidine tract binding protein 1
**Ptger3**	19218	prostaglandin E receptor 3 (subtype EP3)
**Pth1r**	19228	parathyroid hormone 1 receptor
**Ptp4a3**	19245	protein tyrosine phosphatase 4a3
**Ptpre**	19267	protein tyrosine phosphatase, receptor type, E
**Ptprn**	19275	protein tyrosine phosphatase, receptor type, N
**Purb**	19291	purine rich element binding protein B
**Pxylp1**	235534	2-phosphoxylose phosphatase 1
**Pycr1**	209027	pyrroline-5-carboxylate reductase 1
**Qrfpr**	229214	pyroglutamylated RFamide peptide receptor

**R74862**	97423	expressed sequence R74862
**R74862**	.	.
**Rab22a**	19334	RAB22A, member RAS oncogene family
**Rab7**	19349	RAB7, member RAS oncogene family
**Rac3**	170758	RAS-related C3 botulinum substrate 3
**Rad51b**	19363	RAD51 homolog B
**Ralgps2**	78255	Ral GEF with PH domain and SH3 binding motif 2
**Ranbp1**	19385	RAN binding protein 1
**Rapsn**	19400	receptor-associated protein of the synapse
**Rara**	19401	retinoic acid receptor, alpha
**Rarg**	19411	retinoic acid receptor, gamma
**Rasgrp3**	240168	RAS, guanyl releasing protein 3
**Rasl11a**	68895	RAS-like, family 11, member A
**Rassf10**	78748	Ras association (RalGDS/AF-6) domain family (N-terminal) member 10
**Raver2**	242570	ribonucleoprotein, PTB-binding 2
**Rbm12b2**	77604	RNA binding motif protein 12 B2
**Rbm15**	229700	RNA binding motif protein 15
**Rbm27**	225432	RNA binding motif protein 27
**Rbp1**	19659	retinol binding protein 1, cellular
**Rbp7**	63954	retinol binding protein 7, cellular
**Rcn1**	19672	reticulocalbin 1
**Rcsd1**	226594	RCSD domain containing 1
**Reep4**	72549	receptor accessory protein 4
**Rer1**	67830	RER1 retention in endoplasmic reticulum 1 homolog (S. cerevisiae)
**Rfx4**	71137	regulatory factor X, 4 (influences HLA class II expression)
**Rfxank**	19727	regulatory factor X-associated ankyrin-containing protein
**Rgs19**	56470	regulator of G-protein signaling 19
**Rgs7bp**	52882	regulator of G-protein signalling 7 binding protein
**Rhbdf2**	217344	rhomboid 5 homolog 2 (Drosophila)
**Rheb**	19744	Ras homolog enriched in brain
**Ric3**	320360	resistance to inhibitors of cholinesterase 3 homolog (C. elegans)
**Rimbp2**	231760	RIMS binding protein 2
**Rimkla**	194237	ribosomal modification protein rimK-like family member A
**Rims3**	242662	regulating synaptic membrane exocytosis 3
**Ripply2**	382089	ripply2 homolog (zebrafish)
**Rnf152**	320311	ring finger protein 152
**Rnf40**	233900	ring finger protein 40
**Rnh1**	107702	ribonuclease/angiogenin inhibitor 1
**Rnmt**	67897	RNA (guanine-7-) methyltransferase
**Rnps1**	19826	ribonucleic acid binding protein S1
**Robo3**	19649	roundabout homolog 3 (Drosophila)
**Rpl35**	66489	ribosomal protein L35
**Rpl41**	67945	ribosomal protein L41

**Rprml**	104582	reprimo-like
**Rps4x**	20102	ribosomal protein S4, X-linked
**Rps5**	20103	ribosomal protein S5
**Rps6ka2**	20112	ribosomal protein S6 kinase, polypeptide 2
**Rps6ka4**	56613	ribosomal protein S6 kinase, polypeptide 4
**Rps9**	76846	ribosomal protein S9
**Rras**	20130	Harvey rat sarcoma oncogene, subgroup R
**Rsrp1**	27981	arginine/serine rich protein 1
**Rtfdc1**	66404	replication termination factor 2 domain containing 1
**Rtn4r**	65079	reticulon 4 receptor
**Rusc1**	72296	RUN and SH3 domain containing 1
**Rxfp2**	140498	relaxin/insulin-like family peptide receptor 2
**Rxrg**	20183	retinoid X receptor gamma
**Rybp**	56353	RING1 and YY1 binding protein
**S100a1**	20193	S100 calcium binding protein A1
**S100a13**	20196	S100 calcium binding protein A13
**S100a16**	67860	S100 calcium binding protein A16
**S100g**	12309	S100 calcium binding protein G
**Samd14**	217125	sterile alpha motif domain containing 14
**Samd4b**	233033	sterile alpha motif domain containing 4B
**Sap130**	269003	Sin3A associated protein
**Sarnp**	66118	SAP domain containing ribonucleoprotein
**Scaf1**	233208	SR-related CTD-associated factor 1
**Scara5**	71145	scavenger receptor class A, member 5 (putative)
**Scarna17**	100217466	small Cajal body-specific RNA 17
**Scarna17**	.	.
**Scd1**	20249	stearoyl-Coenzyme A desaturase 1
**Scgb1c1**	338417	secretoglobin, family 1C, member 1
**Scn10a**	20264	sodium channel, voltage-gated, type X, alpha
**Scn2b**	72821	sodium channel, voltage-gated, type II, beta
**Scnn1b**	20277	sodium channel, nonvoltage-gated 1 beta
**Sec1**	56546	secretory blood group 1
**Sec24d**	69608	Sec24 related gene family, member D (S. cerevisiae)
**Sec61a1**	53421	Sec61 alpha 1 subunit (S. cerevisiae)
**Selk**	80795	selenoprotein K
**Selplg**	20345	selectin, platelet (p-selectin) ligand
**Sema3f**	20350	sema domain, immunoglobulin domain (Ig), short basic domain, secreted, (semaphorin) 3F
**Sema6c**	20360	sema domain, transmembrane domain (TM), and cytoplasmic domain, (semaphorin) 6C
**Senp8**	71599	SUMO/sentrin specific peptidase 8
**Sep-11**	52398	septin 11
**Serinc2**	230779	serine incorporator 2

**Serpina10**	217847	serine (or cysteine) peptidase inhibitor, clade A (alpha-1 antiproteinase, antitrypsin), member 10
**Serpinf1**	20317	serine (or cysteine) peptidase inhibitor, clade F, member 1
**Serpinh1**	12406	serine (or cysteine) peptidase inhibitor, clade H, member 1
**Setd6**	66083	SET domain containing 6
**Sf3b2**	319322	splicing factor 3b, subunit 2
**Sfn**	55948	stratifin
**Sh2b3**	16923	SH2B adaptor protein 3
**Sh3gl2**	20404	SH3-domain GRB2-like 2
**Sh3kbp1**	58194	SH3-domain kinase binding protein 1
**Shank3**	58234	SH3/ankyrin domain gene 3
**Shroom2**	110380	shroom family member 2
**Skap1**	78473	src family associated phosphoprotein 1
**Slain1**	105439	SLAIN motif family, member 1
**Slamf8**	74748	SLAM family member 8
**Slc11a2**	18174	solute carrier family 11 (proton-coupled divalent metal ion transporters), member 2
**Slc12a5**	57138	solute carrier family 12, member 5
**Slc16a6**	104681	solute carrier family 16 (monocarboxylic acid transporters), member 6
**Slc1a3**	20512	solute carrier family 1 (glial high affinity glutamate transporter), member 3
**Slc25a17**	20524	solute carrier family 25 (mitochondrial carrier, peroxisomal membrane protein), member 17
**Slc25a32**	69906	solute carrier family 25, member 32
**Slc25a45**	107375	solute carrier family 25, member 45
**Slc26a11**	268512	solute carrier family 26, member 11
**Slc2a13**	239606	solute carrier family 2 (facilitated glucose transporter), member 13
**Slc2a3**	20527	solute carrier family 2 (facilitated glucose transporter), member 3
**Slc2a9**	117591	solute carrier family 2 (facilitated glucose transporter), member 9
**Slc30a10**	226781	solute carrier family 30, member 10
**Slc35g2**	245020	solute carrier family 35, member G2
**Slc38a2**	67760	solute carrier family 38, member 2
**Slc38a7**	234595	solute carrier family 38, member 7
**Slc39a9**	328133	solute carrier family 39 (zinc transporter), member 9
**Slc3a1**	20532	solute carrier family 3, member 1
**Slc45a1**	242773	solute carrier family 45, member 1
**Slc45a3**	212980	solute carrier family 45, member 3
**Slc48a1**	67739	solute carrier family 48 (heme transporter), member 1
**Slc4a1**	20533	solute carrier family 4 (anion exchanger), member 1
**Slc52a3**	69698	solute carrier protein family 52, member 3
**Slc5a3**	53881	solute carrier family 5 (inositol transporters), member 3
**Slc6a15**	103098	solute carrier family 6 (neurotransmitter transporter), member 15
**Slc6a2**	20538	solute carrier family 6 (neurotransmitter transporter, noradrenalin), member 2
**Slc7a1**	11987	solute carrier family 7 (cationic amino acid transporter, y+ system), member 1

**Slc9a5**	277973	solute carrier family 9 (sodium/hydrogen exchanger), member 5
**Slpi**	20568	secretory leukocyte peptidase inhibitor
**Smad5**	17129	SMAD family member 5
**Smarcd1**	83797	SWI/SNF related, matrix associated, actin dependent regulator of chromatin, subfamily d, member 1
**Smco3**	654818	single-pass membrane protein with coiled-coil domains 3
**Smg5**	229512	Smg-5 homolog, nonsense mediated mRNA decay factor (C. elegans)
**Smg9**	71997	smg-9 homolog, nonsense mediated mRNA decay factor (C. elegans)
**Smpd3**	58994	sphingomyelin phosphodiesterase 3, neutral
**Smtnl1**	68678	smoothelin-like 1
**Snf8**	27681	SNF8, ESCRT-II complex subunit, homolog (S. cerevisiae)
**Snhg12**	100039864	small nucleolar RNA host gene 12
**Snhg12**	.	.
**Snhg7**	72091	small nucleolar RNA host gene 7
**Snhg7**	.	.
**Snip1**	76793	Smad nuclear interacting protein 1
**Snora16a**	100310813	small nucleolar RNA, H/ACA box 16A
**Snora16a**	.	.
**Snora17**	.	.
**Snora17**	.	.
**Snora17**	.	.
**Snora17**	.	.
**Snora17**	.	.
**Snora17**	.	.
**Snora17**	100306954	small nucleolar RNA, H/ACA box 17
**Snora43**	.	.
**Snora43**	.	.
**Snora43**	.	.
**Snora43**	.	.
**Snora43**	.	.
**Snora43**	100306955	small nucleolar RNA, H/ACA box 43
**Snora44**	.	.
**Snora44**	100217418	small nucleolar RNA, H/ACA box 44
**Snora47**	100217450	small nucleolar RNA, H/ACA box 47
**Snora47**	.	.
**Snora61**	100217440	small nucleolar RNA, H/ACA box 61
**Snora61**	.	.
**Snord99**	.	.
**Snord99**	100217437	small nucleolar RNA, C/D box 99
**Sox21**	223227	SRY (sex determining region Y)-box 21
**Sp2**	78912	Sp2 transcription factor
**Sp7**	170574	Sp7 transcription factor 7
**Sp8**	320145	trans-acting transcription factor 8

**Spen**	56381	SPEN homolog, transcriptional regulator (Drosophila)
**Spn-ps**	20738	sialophorin, pseudogene
**Spns2**	216892	spinster homolog 2
**Spo11**	26972	SPO11 meiotic protein covalently bound to DSB homolog (S. cerevisiae)
**Sppl3**	74585	signal peptide peptidase 3
**Spz1**	79401	spermatogenic leucine zipper 1
**Srcin1**	56013	SRC kinase signaling inhibitor 1
**Srek1ip1**	67288	splicing regulatory glutamine/lysine-rich protein 1interacting protein 1
**Srrm1**	51796	serine/arginine repetitive matrix 1
**St6galnac3**	20447	ST6 (alpha-N-acetyl-neuraminyl-2,3-beta-galactosyl-1,3)-N-acetylgalactosaminide alpha-2,6-sialyltransferase 3
**Stambp**	70527	STAM binding protein
**Star**	20845	steroidogenic acute regulatory protein
**Ston2**	108800	stonin 2
**Stpg1**	78806	sperm tail PG rich repeat containing 1
**Strip2**	320609	striatin interacting protein 2
**Suclg2**	20917	succinate-Coenzyme A ligase, GDP-forming, beta subunit
**Supt4a**	20922	suppressor of Ty 4A
**Susd4**	96935	sushi domain containing 4
**Susd5**	382111	sushi domain containing 5
**Svop**	68666	SV2 related protein
**Syce2**	71846	synaptonemal complex central element protein 2
**Syce3**	75459	synaptonemal complex central element protein 3
**Sycp1**	20957	synaptonemal complex protein 1
**Syne2**	319565	spectrin repeat containing, nuclear envelope 2
**Syne3**	212073	spectrin repeat containing, nuclear envelope family member 3
**Syngr1**	20972	synaptogyrin 1
**Syt14**	329324	synaptotagmin XIV
**Syt17**	110058	synaptotagmin XVII
**Syt9**	60510	synaptotagmin IX
**Sytl1**	269589	synaptotagmin-like 1
**Sytl2**	83671	synaptotagmin-like 2
**Tac4**	93670	tachykinin 4
**Taf10**	24075	TAF10 RNA polymerase II, TATA box binding protein (TBP)-associated factor
**Taf8**	63856	TAF8 RNA polymerase II, TATA box binding protein (TBP)-associated factorq
**Taldo1**	21351	transaldolase 1
**Tapbp**	21356	TAP binding protein
**Tbc1d22a**	223754	TBC1 domain family, member 22a
**Tbc1d9**	71310	TBC1 domain family, member 9
**Tbce**	70430	tubulin-specific chaperone E
**Tbkbp1**	73174	TBK1 binding protein 1
**Tbrg4**	21379	transforming growth factor beta regulated gene 4

**Tbx19**	83993	T-box 19
**Tbx22**	245572	T-box 22
**Tbx3os2**	.	.
**Tbx3os2**	433944	T-box 3, opposite strand 2
**Tceal5**	331532	transcription elongation factor A (SII)-like 5
**Tceal8**	66684	transcription elongation factor A (SII)-like 8
**Tcirg1**	27060	T cell, immune regulator 1, ATPase, H+ transporting, lysosomal V0 protein A3
**Tcp1**	21454	t-complex protein 1
**Tctex1d1**	67344	Tctex1 domain containing 1
**Tctex1d4**	242646	Tctex1 domain containing 4
**Tdp2**	56196	tyrosyl-DNA phosphodiesterase 2
**Tead1**	21676	TEA domain family member 1
**Tesc**	57816	tescalcin
**Tet2**	214133	tet methylcytosine dioxygenase 2
**Tex13a**	67944	testis expressed 13A
**Tfb2m**	15278	transcription factor B2, mitochondrial
**Tfcp2l1**	81879	transcription factor CP2-like 1
**Tgfb1i1**	21804	transforming growth factor beta 1 induced transcript 1
**Tgif1**	21815	TGFB-induced factor homeobox 1
**Tgoln1**	22134	trans-golgi network protein
**Tgoln2**	22135	trans-golgi network protein 2
**Tgtp1**	21822	T cell specific GTPase 1
**Tgtp2**	100039796	T cell specific GTPase 2
**Thbs2**	21826	thrombospondin 2
**Ticam1**	106759	toll-like receptor adaptor molecule 1
**Timm10**	30059	translocase of inner mitochondrial membrane 10
**Timp3**	21859	tissue inhibitor of metalloproteinase 3
**Tmed1**	17083	transmembrane emp24 domain containing 1
**Tmem100**	67888	transmembrane protein 100
**Tmem116**	77462	transmembrane protein 116
**Tmem120a**	215210	transmembrane protein 120A
**Tmem121**	69195	transmembrane protein 121
**Tmem132a**	98170	transmembrane protein 132A
**Tmem132cos**	67351	transmembrane protein 132C, opposite strand
**Tmem132cos**	.	.
**Tmem132d**	243274	transmembrane protein 132D
**Tmem138**	72982	transmembrane protein 138
**Tmem177**	66343	transmembrane protein 177
**Tmem179**	104885	transmembrane protein 179
**Tmem258**	69038	transmembrane protein 258
**Tmem259**	216157	transmembrane protein 259
**Tmem51**	214359	transmembrane protein 51

**Tmem51os1**	100038693	Tmem51 opposite strand 1
**Tmem51os1**	.	.
**Tmem54**	66260	transmembrane protein 54
**Tmem67**	329795	transmembrane protein 67
**Tmem87a**	211499	transmembrane protein 87A
**Tmem88b**	320587	transmembrane protein 88B
**Tmprss12**	75002	transmembrane (C-terminal) protease, serine 12
**Tmsb4x**	19241	thymosin, beta 4, X chromosome
**Tmtc2**	278279	transmembrane and tetratricopeptide repeat containing 2
**Tmx2**	66958	thioredoxin-related transmembrane protein 2
**Tnfrsf9**	21942	tumor necrosis factor receptor superfamily, member 9
**Tnnc1**	21924	troponin C, cardiac/slow skeletal
**Tpcn2**	233979	two pore segment channel 2
**Tpp1**	12751	tripeptidyl peptidase I
**Tppp3**	67971	tubulin polymerization-promoting protein family member 3
**Tpra1**	24100	transmembrane protein, adipocyte asscociated 1
**Traf7**	224619	TNF receptor-associated factor 7
**Traip**	22036	TRAF-interacting protein
**Tram1l1**	229801	translocation associated membrane protein 1-like 1
**Trap1a**	22037	tumor rejection antigen P1A
**Trappc8**	75964	trafficking protein particle complex 8
**Trib3**	228775	tribbles homolog 3 (Drosophila)
**Trim46**	360213	tripartite motif-containing 46
**Trim52**	212085	tripartite motif-containing 52
**Trim67**	330863	tripartite motif-containing 67
**Trnp1**	69539	TMF1-regulated nuclear protein 1
**Troap**	78733	trophinin associated protein
**Tsc22d4**	78829	TSC22 domain family, member 4
**Tspan11**	68498	tetraspanin 11
**Tspan18**	241556	tetraspanin 18
**Tspan31**	67125	tetraspanin 31
**Tsr1**	104662	TSR1 20S rRNA accumulation
**Tssk3**	58864	testis-specific serine kinase 3
**Tsx**	22127	testis specific X-linked gene
**Ttc25**	74407	tetratricopeptide repeat domain 25
**Ttll9**	74711	tubulin tyrosine ligase-like family, member 9
**Tuba8**	53857	tubulin, alpha 8
**Tut1**	70044	terminal uridylyl transferase 1, U6 snRNA-specific
**Txlng**	353170	taxilin gamma
**Tyw1**	100929	tRNA-yW synthesizing protein 1 homolog (S. cerevisiae)
**Ubap2l**	74383	ubiquitin-associated protein 2-like
**Ubox5**	140629	U box domain containing 5
**Ucp2**	22228	uncoupling protein 2 (mitochondrial, proton carrier)

**Ufc1**	66155	ubiquitin-fold modifier conjugating enzyme 1
**Unc5c**	22253	unc-5 homolog C (C. elegans)
**Upp2**	76654	uridine phosphorylase 2
**Urad**	231903	ureidoimidazoline (2-oxo-4-hydroxy-4-carboxy-5) decarboxylase
**Urb1**	207932	URB1 ribosome biogenesis 1 homolog (S. cerevisiae)
**Usp12**	22217	ubiquitin specific peptidase 12
**Usp19**	71472	ubiquitin specific peptidase 19
**Usp35**	244144	ubiquitin specific peptidase 35
**Usp5**	22225	ubiquitin specific peptidase 5 (isopeptidase T)
**Utp3**	65961	UTP3, small subunit (SSU) processome component, homolog (S. cerevisiae)
**Vac14**	234729	Vac14 homolog (S. cerevisiae)
**Vash1**	238328	vasohibin 1
**Vdr**	22337	vitamin D receptor
**Vmn2r9**	435864	vomeronasal 2, receptor 9
**Vrk1**	22367	vaccinia related kinase 1
**Vsig1**	78789	V-set and immunoglobulin domain containing 1
**Vsig8**	240916	V-set and immunoglobulin domain containing 8
**Vstm2l**	277432	V-set and transmembrane domain containing 2-like
**Vwa1**	246228	von Willebrand factor A domain containing 1
**Vwf**	22371	Von Willebrand factor homolog
**Wbscr17**	212996	Williams-Beuren syndrome chromosome region 17 homolog (human)
**Wbscr28**	76629	Williams-Beuren syndrome chromosome region 28 (human)
**Wdr34**	71820	WD repeat domain 34
**Wdr46**	57315	WD repeat domain 46
**Wdr55**	67936	WD repeat domain 55
**Wdr81**	192652	WD repeat domain 81
**Wfdc11**	629761	WAP four-disulfide core domain 11
**Wfikkn2**	278507	WAP, follistatin/kazal, immunoglobulin, kunitz and netrin domain containing 2
**Wipf1**	215280	WAS/WASL interacting protein family, member 1
**Wnt4**	22417	wingless-type MMTV integration site family, member 4
**Wnt7a**	22421	wingless-type MMTV integration site family, member 7A
**Wscd2**	320916	WSC domain containing 2
**Xbp1**	22433	X-box binding protein 1
**Xpo5**	72322	exportin 5
**Zbtb22**	81630	zinc finger and BTB domain containing 22
**Zbtb40**	230848	zinc finger and BTB domain containing 40
**Zbtb46**	72147	zinc finger and BTB domain containing 46
**Zbtb8a**	73680	zinc finger and BTB domain containing 8a
**Zc3h10**	103284	zinc finger CCCH type containing 10
**Zc3hc1**	232679	zinc finger, C3HC type 1
**Zcwpw1**	381678	zinc finger, CW type with PWWP domain 1
**Zdhhc4**	72881	zinc finger, DHHC domain containing 4

**Zfp128**	243833	zinc finger protein 128
**Zfp219**	69890	zinc finger protein 219
**Zfp286**	192651	zinc finger protein 286
**Zfp318**	57908	zinc finger protein 318
**Zfp384**	269800	zinc finger protein 384
**Zfp420**	233058	zinc finger protein 420
**Zfp455**	218311	zinc finger protein 455
**Zfp456**	408065	zinc finger protein 456
**Zfp51**	22709	zinc finger protein 51
**Zfp536**	243937	zinc finger protein 536
**Zfp54**	22712	zinc finger protein 54
**Zfp58**	238693	zinc finger protein 58
**Zfp593**	68040	zinc finger protein 593
**Zfp608**	269023	zinc finger protein 608
**Zfp612**	234725	zinc finger protein 612
**Zfp641**	239652	zinc finger protein 641
**Zfp646**	233905	zinc finger protein 646
**Zfp653**	319601	zinc finger protein 653
**Zfp668**	244219	zinc finger protein 668
**Zfp740**	68744	zinc finger protein 740
**Zfp759**	268670	zinc finger protein 759
**Zfp760**	240034	zinc finger protein 760
**Zfp78**	330463	zinc finger protein 78
**Zfp874b**	408067	zinc finger protein 874b
**Zfp935**	71508	zinc finger protein 935
**Zfp940**	233057	zinc finger protein 940
**Zfp945**	240041	zinc finger protein 945
**Zfp949**	71640	zinc finger protein 949
**Zfpl1**	81909	zinc finger like protein 1
**Zfyve26**	211978	zinc finger, FYVE domain containing 26
**Zic1**	22771	zinc finger protein of the cerebellum 1
**Zic4**	22774	zinc finger protein of the cerebellum 4
**Zmynd8**	228880	zinc finger, MYND-type containing 8
**Znhit1**	70103	zinc finger, HIT domain containing 1
**Zscan22**	232878	zinc finger and SCAN domain containing 22
**Zxda**	.	.
**Zxda**	668171	zinc finger, X-linked, duplicated A
**Zyg11a**	230590	zyg-11 family member A, cell cycle regulator

Table S4: Summary table reporting fold changes and p-values of RCF vs. CT contrasts for SAGE and all ChIP-seq experiments. Table reports only genes that could be analyzed at trascriptome and for least one histone mark.

Gene Symbol	FC SAGE	FC H3K4me3	FC H3Ac	FC H3K27me3	p.value SAGE	p.value H3K4me3	p.value H3AC	p.value H3K27me3
**Scaf1**	6.10E-01	1.79E+00	.	.	4.88E-01	3.39E-13	.	.
**Pcp4**	-5.86E-01	.	.	1.26E+00	2.66E-01	.	.	5.47E-05
**Clptm1**	1.36E-01	.	1.48E+00	.	6.93E-01	.	7.59E-08	.
**Ano2**	-1.16E+00	.	.	1.01E+00	1.62E-01	.	.	1.77E-07
**Pcyt1b**	-4.72E-01	.	.	2.41E+00	3.32E-01	.	.	1.49E-19
**Cpeb1**	5.80E-02	.	.	-1.02E+00	9.67E-01	.	.	3.20E-10
**Cbln1**	-1.36E+00	.	.	2.87E+00	4.60E-02	.	.	1.37E-08
**Ngdn**	9.95E-01	.	1.22E+00	.	1.91E-01	.	1.96E-09	.
**Efna1**	2.82E-01	.	.	-1.02E+00	5.50E-01	.	.	4.14E-07
**Clec16a**	1.31E-01	.	1.36E+00	.	6.70E-01	.	1.78E-07	.
**Med20**	9.22E-01	.	1.62E+00	.	1.33E-01	.	5.61E-12	.
**Maf**	1.73E-01	.	.	-1.19E+00	6.46E-01	.	.	7.07E-15
**Clstn3**	3.03E-01	2.21E+00	1.66E+00	.	4.32E-01	3.38E-21	3.07E-14	.
**Dhx8**	8.52E-01	1.07E+00	.	.	1.15E-01	1.84E-07	.	.
**Shank3**	2.93E-01	.	.	-1.88E+00	5.28E-01	.	.	1.04E-06
**Mospd3**	7.52E-01	.	1.32E+00	.	3.20E-01	.	1.88E-07	.
**B4galnt1**	5.19E-01	.	.	-1.43E+00	3.39E-01	.	.	5.98E-10
**Slc25a45**	4.09E-01	.	1.75E+00	.	5.69E-01	.	5.47E-10	.
**Arsg**	3.43E-01	.	.	-1.29E+00	4.47E-01	.	.	6.61E-03
**Ptp4a3**	4.01E-01	.	1.01E+00	.	5.24E-01	.	2.42E-04	.
**Sox21**	-3.95E-01	.	.	3.02E+00	3.88E-01	.	.	1.84E-15
**1700011A 15Rik**	-5.50E-02	.	.	3.01E+00	9.31E-01	.	.	3.11E-08
**Zfp384**	8.15E-01	.	1.04E+00	.	1.58E-01	.	8.15E-07	.
**Zfp935**	7.35E-01	.	3.13E+00	.	3.39E-01	.	4.92E-05	.
**Fzd7**	6.40E-02	.	.	-1.02E+00	8.80E-01	.	.	2.45E-16
**Gpsm1**	4.00E-02	.	1.24E+00	.	9.53E-01	.	1.72E-06	.
**Ucp2**	-3.09E-01	.	.	2.47E+00	6.22E-01	.	.	2.58E-03
**Rad51b**	3.03E+00	.	1.56E+00	.	4.14E-03	.	2.46E-03	.
**Msrb1**	3.32E-01	.	1.72E+00	.	6.15E-01	.	4.97E-06	.
**Smg9**	1.04E+00	.	1.97E+00	.	8.45E-02	.	7.53E-04	.
**Nt5e**	7.40E-02	.	.	-1.01E+00	9.45E-01	.	.	2.93E-04
**Dnajc6**	4.30E-02	.	.	-1.12E+00	9.23E-01	.	.	1.47E-03
**Dctn2**	1.24E-01	.	.	-1.39E+00	7.00E-01	.	.	2.79E-06
**Ccdc174**	-4.80E-02	.	.	1.15E+00	8.83E-01	.	.	3.90E-07

**Cd164**	1.03E+00	1.36E+00	.	.	8.30E-02	1.56E-12	.	.
**2410124H12Rik**	-2.10E-02	.	.	1.43E+00	9.82E-01	.	.	8.16E-10
**Ech1**	1.76E-01	.	1.11E+00	.	7.32E-01	.	4.04E-08	.
**Lmtk2**	-3.80E-02	.	.	2.29E+00	9.05E-01	.	.	4.08E-10
**Pafah2**	1.40E-01	.	1.12E+00	.	7.75E-01	.	7.25E-06	.
**Vrk1**	-2.09E-01	.	.	1.23E+00	7.32E-01	.	.	4.79E-09
**Lrig2**	5.30E-01	.	1.76E+00	.	4.36E-01	.	4.06E-08	.
**P2rx7**	8.43E-01	.	1.34E+00	.	5.53E-01	.	1.84E-03	.
**E130006D01Rik**	2.18E+00	.	1.92E+00	1.02E+00	2.52E-02	.	3.05E-07	4.71E-12
**Frmd6**	5.24E-01	1.01E+00	.	.	2.05E-01	8.09E-06	.	.
**Cecr2**	9.09E-01	.	.	-1.56E+00	1.24E-01	.	.	3.56E-04
**Mdm1**	-1.31E+00	.	.	7.33E+00	6.59E-02	.	.	3.64E-12
**Tceal5**	-3.77E-01	.	.	3.57E+00	2.23E-01	.	.	2.60E-13
**Ergic2**	6.90E-02	.	2.15E+00	.	8.33E-01	.	6.93E-07	.
**Pde3a**	1.34E+00	.	.	-1.10E+00	1.16E-01	.	.	8.50E-10
**Map3k1**	9.70E-01	.	.	-1.46E+00	1.62E-01	.	.	2.46E-03
**Snora17**	1.54E+00	1.21E+00	.	.	1.12E-01	2.60E-09	.	.
**Elavl4**	2.94E-01	.	.	-1.05E+00	3.94E-01	.	.	3.26E-04
**Slc7a1**	1.99E-01	.	.	-1.32E+00	6.33E-01	.	.	1.84E-03
**Msrb3**	-1.60E-02	.	.	3.42E+00	9.72E-01	.	.	1.42E-13
**Pacsin3**	2.28E-01	.	.	-1.06E+00	6.75E-01	.	.	6.45E-06
**Cenpu**	1.52E+00	2.52E+00	.	.	1.18E-01	3.14E-19	.	.
**Pdia4**	3.19E-01	.	1.40E+00	.	6.11E-01	.	6.43E-03	.
**Nlrp6**	-2.52E-01	.	.	1.31E+00	7.01E-01	.	.	4.89E-07
**Arhgap26**	7.00E-02	1.26E+00	1.03E+00	.	8.65E-01	4.23E-07	1.85E-03	.
**Zfp760**	7.00E-02	2.27E+00	1.46E+00	.	8.61E-01	1.39E-20	1.61E-11	.
**Eif4e3**	2.16E-01	.	.	-1.15E+00	6.87E-01	.	.	2.63E-03
**Gm7538**	-5.32E-01	.	.	1.04E+00	4.58E-01	.	.	2.75E-06
**Gmfg**	2.55E-01	2.44E+00	.	.	6.69E-01	6.35E-22	.	.
**Pls1**	3.90E-02	.	.	-1.48E+00	9.26E-01	.	.	2.64E-04
**Zfp455**	1.22E+00	.	4.41E+00	.	2.73E-01	.	1.52E-11	.
**Mmd**	3.34E-01	.	.	-1.05E+00	4.24E-01	.	.	7.15E-03
**Ezh1**	-3.71E-01	.	.	1.74E+00	7.24E-01	.	.	1.34E-08
**Emb**	3.86E-01	.	.	-1.13E+00	4.50E-01	.	.	8.90E-09
**Negr1**	1.79E-01	.	.	-1.15E+00	5.90E-01	.	.	1.02E-04
**Tbkbp1**	5.39E-01	.	2.37E+00	-1.36E+00	2.38E-01	.	5.54E-06	6.47E-05
**Brip1**	1.51E-01	.	1.69E+00	.	7.03E-01	.	2.93E-05	.
**Zfp420**	4.45E-01	1.00E+00	.	.	4.70E-01	5.70E-06	.	.
**Aco2**	1.76E-01	.	1.00E+00	.	6.24E-01	.	8.56E-07	.
**Litaf**	-2.56E-01	.	-1.65E+00	.	5.48E-01	.	6.10E-10	.
**Stpg1**	2.54E-01	.	1.38E+00	.	7.15E-01	.	7.22E-07	.

**Xbp1**	2.20E-01	1.03E+00	.	.	5.89E-01	1.33E-04	.	.
**Atg4a**	-1.13E-01	.	.	1.11E+00	7.78E-01	.	.	1.77E-06
**A330070 K13Rik**	2.70E-02	.	1.18E+00	.	9.55E-01	.	2.19E-03	.
**Psmd10**	-5.55E-01	.	.	1.11E+00	2.69E-01	.	.	1.77E-06
**Cdv3**	4.69E-01	.	.	-1.49E+00	2.54E-01	.	.	7.71E-08
**Rnps1**	7.86E-01	.	1.17E+00	.	1.09E-01	.	1.05E-03	.
**Spns2**	5.00E-01	.	1.90E+00	.	5.36E-01	.	1.29E-06	.
**Pycr1**	6.85E-01	.	.	-1.12E+00	3.77E-01	.	.	1.75E-07
**Zic4**	-1.21E+00	.	.	1.93E+00	1.06E-01	.	.	1.25E-18
**Gabrd**	-1.02E+00	.	-1.26E+00	.	1.62E-01	.	1.41E-05	.
**Pptc7**	2.10E-02	.	1.85E+00	.	9.59E-01	.	4.36E-09	.
**Abhd4**	1.38E+00	1.23E+00	.	.	1.06E-01	1.68E-12	.	.
**Mxd4**	3.21E-01	.	1.10E+00	.	4.49E-01	.	1.61E-03	.
**Daam2**	1.68E-01	.	1.47E+00	.	6.52E-01	.	1.81E-08	.
**Cldn15**	3.78E-01	.	2.65E+00	.	7.11E-01	.	5.67E-12	.
**Slc48a1**	1.88E-01	.	1.38E+00	.	6.49E-01	.	1.12E-05	.
**Slc2a9**	1.62E-01	1.57E+00	.	.	8.61E-01	2.91E-09	.	.
**8430408G 22Rik**	6.85E-01	.	.	-1.23E+00	3.77E-01	.	.	5.07E-06
**Drg2**	5.47E-01	1.11E+00	.	.	2.41E-01	4.17E-11	.	.
**D430020J 02Rik**	6.56E-01	.	1.05E+00	.	4.30E-01	.	2.04E-04	.
**Rbm15**	1.80E+00	.	.	-1.82E+00	6.56E-02	.	.	3.88E-04
**Akr1b10**	4.86E-01	1.68E+00	.	.	3.59E-01	9.34E-12	.	.
**Pgbd5**	1.89E-01	.	.	-1.39E+00	5.96E-01	.	.	4.97E-04
**Scnn1b**	-1.07E+00	.	.	2.34E+00	3.30E-01	.	.	3.39E-08
**Usp19**	3.52E-01	.	.	-1.07E+00	4.70E-01	.	.	8.44E-10
**Bmpr1b**	-1.44E+00	.	.	1.26E+00	4.95E-02	.	.	6.48E-05
**Cox6b1**	7.22E-01	.	1.07E+00	.	3.88E-01	.	1.54E-05	.
**Cldn2**	-6.00E-02	.	.	3.14E+00	9.21E-01	.	.	1.64E-12
**Tspan11**	-2.63E-01	.	.	1.00E+00	7.23E-01	.	.	3.11E-16
**Lin28a**	-5.58E-01	.	.	1.52E+00	5.05E-01	.	.	2.53E-06
**9430038I0 1Rik**	1.02E+00	1.22E+00	.	.	1.03E-01	8.29E-06	.	.
**Mgat3**	1.81E-01	.	1.87E+00	.	7.22E-01	.	2.08E-08	.
**Faim2**	7.73E-01	.	.	-1.29E+00	7.92E-02	.	.	4.41E-04
**Nr5a2**	-5.32E-01	.	.	3.69E+00	4.58E-01	.	.	1.63E-06
**Gpr3**	1.19E+00	.	.	-1.06E+00	5.40E-01	.	.	5.28E-07
**Frmd8**	1.35E+00	.	1.75E+00	.	2.63E-01	.	5.47E-10	.
**Dyrk3**	8.87E-01	.	.	-1.28E+00	1.35E-01	.	.	4.60E-03
**Auts2**	5.21E-01	.	.	-1.14E+00	5.89E-01	.	.	3.06E-04
**Ccdc60**	-6.42E-01	.	.	1.10E+00	5.04E-01	.	.	3.23E-13
**Fis1**	5.75E-01	.	2.65E+00	.	3.46E-01	.	5.67E-12	.

**Mpv17**	4.76E-01	1.02E+00	.	.	3.01E-01	1.43E-06	.	.
**Lrrn1**	4.00E-01	.	.	-1.75E+00	5.05E-01	.	.	4.18E-09
**Taf8**	3.70E-01	1.50E+00	.	.	6.85E-01	1.23E-21	.	.
**Traip**	1.12E+00	.	.	-1.20E+00	2.11E-01	.	.	2.75E-08
**P2ry2**	-1.59E-01	.	.	2.16E+00	8.09E-01	.	.	8.60E-09
**Maged1**	-2.06E-01	.	.	3.40E+00	5.60E-01	.	.	1.62E-14
**Ubap2l**	7.57E-01	.	1.54E+00	.	3.70E-01	.	3.73E-08	.
**Tmsb4x**	-6.10E-02	.	.	1.38E+00	8.98E-01	.	.	6.73E-06
**Cnn3**	5.56E-01	.	.	-2.25E+00	2.36E-01	.	.	9.26E-05
**Tsc22d4**	2.40E+00	.	.	-1.28E+00	7.80E-04	.	.	9.28E-05
**Kcnd3os**	1.40E+00	.	.	-1.24E+00	8.76E-02	.	.	2.92E-04
**Iqsec1**	3.13E-01	.	1.93E+00	.	3.71E-01	.	1.42E-04	.
**Slc26a11**	2.80E-01	.	.	-1.76E+00	4.47E-01	.	.	2.19E-06
**Mapkapk 3**	8.41E-01	.	.	-1.99E+00	3.01E-01	.	.	3.30E-09
**Fbln5**	-6.49E-01	.	.	5.80E+00	4.27E-01	.	.	5.44E-20
**Irgm1**	2.25E-01	.	1.92E+00	.	5.70E-01	.	5.50E-09	.
**Aqp2**	1.12E+00	.	.	-1.21E+00	2.67E-01	.	.	1.01E-04
**Gm17296**	9.05E-01	.	.	-1.18E+00	2.63E-01	.	.	5.35E-07
**Junb**	2.33E-01	.	.	-2.12E+00	8.80E-01	.	.	4.59E-17
**B3glct**	2.15E-01	.	.	-1.51E+00	6.34E-01	.	.	3.86E-04
**Plk2**	1.37E-01	.	.	-1.57E+00	8.05E-01	.	.	2.93E-04
**Ifitm10**	1.24E+00	.	.	-1.22E+00	1.31E-01	.	.	2.00E-09
**Mtmr4**	1.08E+00	.	1.66E+00	.	1.90E-01	.	9.94E-10	.
**Wdr55**	1.29E-01	1.73E+00	.	.	7.92E-01	2.13E-20	.	.
**Ntm**	4.43E-01	1.53E+00	.	.	2.67E-01	6.11E-15	.	.
**Chml**	4.34E-01	.	.	-1.52E+00	2.95E-01	.	.	9.04E-03
**Pparg**	1.27E+00	.	.	-1.03E+00	3.81E-01	.	.	6.03E-10
**Epb4.1**	1.50E-01	.	.	-2.23E+00	8.78E-01	.	.	1.92E-04
**Rfxank**	9.00E-02	2.58E+00	1.31E+00	.	9.36E-01	3.44E-25	3.62E-10	.
**Gnai3**	2.53E-01	.	.	-1.21E+00	6.55E-01	.	.	1.31E-05
**Fam19a5**	1.52E-01	.	.	-1.20E+00	7.97E-01	.	.	6.43E-04
**Rsrp1**	3.46E-01	.	1.00E+00	.	4.63E-01	.	1.39E-03	.
**Pars2**	1.66E-01	1.03E+00	.	2.60E+00	7.65E-01	5.94E-08	.	1.94E-21
**Nuak2**	8.61E-01	.	.	-2.59E+00	4.02E-01	.	.	3.40E-05
**Setd6**	2.10E+00	.	.	-1.00E+00	1.08E-01	.	.	5.77E-07
**AU040320**	1.80E-02	.	1.05E+00	.	9.80E-01	.	3.91E-06	.
**Brd8**	1.80E-02	1.26E+00	.	.	9.63E-01	2.59E-08	.	.
**Banp**	1.30E+00	.	1.08E+00	.	6.70E-02	.	4.50E-05	.
**Srcin1**	1.94E-01	.	.	-1.13E+00	6.22E-01	.	.	2.56E-04
**Elavl2**	-6.80E-02	.	.	1.33E+00	8.34E-01	.	.	1.01E-07
**Cap1**	1.68E-01	.	2.66E+00	.	6.85E-01	.	5.67E-11	.
**Fjx1**	1.35E-01	.	.	-1.19E+00	9.44E-01	.	.	4.16E-06

**Amn**	2.75E-01	.	.	-1.14E+00	6.48E-01	.	.	2.94E-10
**Rps6ka2**	4.85E-01	1.53E+00	.	.	3.93E-01	3.14E-14	.	.
**Ccdc93**	9.40E-01	.	.	-1.06E+00	1.14E-01	.	.	2.19E-03
**Bach1**	1.60E-02	.	1.60E+00	.	9.67E-01	.	3.80E-06	.
**Zfp456**	2.76E-01	.	1.49E+00	.	6.79E-01	.	2.87E-04	.
**Hook1**	2.58E-01	.	.	-1.25E+00	7.00E-01	.	.	2.09E-06
**Scd1**	3.23E-01	.	1.54E+00	.	3.46E-01	.	8.44E-07	.
**Btbd19**	1.04E+00	.	1.40E+00	.	3.71E-01	.	1.02E-07	.
**Car5a**	3.80E-02	.	1.08E+00	.	9.65E-01	.	4.50E-05	.
**Samd4b**	3.84E-01	2.44E+00	.	.	5.47E-01	6.35E-22	.	.
**Rtn4r**	-2.05E-01	.	- 1.22E+00	.	5.84E-01	.	2.24E-04	.
**Ier2**	1.90E-01	2.57E+00	1.04E+00	-1.05E+00	6.44E-01	1.82E-26	3.69E-08	8.51E-10
**Itpripl2**	-1.25E-01	.	.	2.65E+00	8.05E-01	.	.	1.71E-09
**Magi3**	1.50E-01	.	.	-1.38E+00	7.31E-01	.	.	1.26E-04
**Mfsd5**	4.35E-01	.	1.48E+00	.	2.69E-01	.	4.32E-06	.
**Mc3r**	-8.40E-01	.	.	1.16E+00	4.27E-01	.	.	6.46E-05
**Rnf40**	2.10E-01	.	1.12E+00	.	6.90E-01	.	1.16E-04	.
**Gjb5**	9.95E-01	.	.	-1.19E+00	1.91E-01	.	.	1.09E-03
**Awat2**	-1.69E+00	.	.	2.99E+00	6.16E-02	.	.	1.08E-15
**Disp2**	4.60E-01	1.12E+00	.	.	3.08E-01	4.42E-05	.	.
**Tmem258**	9.95E-01	1.89E+00	1.01E+00	.	1.91E-01	1.89E-14	2.91E-04	.
**Iqsec2**	-1.29E+00	.	.	1.12E+00	8.25E-02	.	.	1.80E-07
**Smad5**	8.73E-01	.	1.22E+00	.	5.33E-02	.	3.13E-04	.
**Kif20a**	6.34E-01	1.26E+00	.	.	4.22E-01	2.59E-08	.	.
**Sppl3**	3.90E-02	.	.	-1.04E+00	9.05E-01	.	.	5.92E-03
**Smg5**	1.44E-01	.	.	-1.51E+00	7.95E-01	.	.	1.27E-05
**Ppp4r1l-ps**	4.58E-01	1.03E+00	.	.	3.79E-01	1.98E-05	.	.
**Tmem177**	1.71E-01	.	.	-1.53E+00	6.92E-01	.	.	3.89E-05
**Zfp759**	4.59E-01	.	2.52E+00	.	6.01E-01	.	8.97E-06	.
**Nfib**	5.31E-01	.	1.18E+00	.	2.53E-01	.	7.79E-03	.
**Fam171a1**	-4.62E-01	.	- 1.04E+00	.	4.23E-01	.	4.07E-03	.
**S100a16**	6.20E-02	.	1.62E+00	.	9.06E-01	.	4.34E-07	.
**Vwa1**	7.80E-01	.	.	-2.96E+00	1.88E-01	.	.	4.03E-04
**Gpc6**	1.47E-01	.	.	-1.08E+00	8.25E-01	.	.	6.42E-09
**Rbm27**	1.40E-02	.	1.35E+00	.	9.67E-01	.	3.81E-05	.
**Slc11a2**	5.72E-01	1.23E+00	.	.	6.96E-01	1.20E-16	.	.
**1700025G04Rik**	2.13E-01	.	.	-1.33E+00	5.68E-01	.	.	7.92E-03
**Clec4a1**	4.62E-01	.	.	-1.12E+00	7.46E-01	.	.	1.25E-04
**Zfp653**	3.64E-01	.	1.42E+00	.	2.74E-01	.	9.67E-07	.
**Oasl1**	1.61E+00	.	.	-1.64E+00	2.51E-01	.	.	5.78E-05
**Igfals**	7.16E-01	.	1.19E+00	.	4.66E-01	.	1.07E-03	.

**Zfp51**	6.40E-01	1.11E+00	.	.	2.90E-01	1.05E-12	.	.
**Eif4b**	-1.74E-01	.	.	3.59E+00	7.55E-01	.	.	1.97E-11
**Rasgrp3**	7.40E-02	.	2.07E+00	.	8.83E-01	.	1.24E-08	.
**Cdh4**	-1.20E-01	.	.	1.38E+00	7.88E-01	.	.	8.57E-10
**Ap5z1**	3.20E-01	.	1.35E+00	.	5.94E-01	.	5.52E-03	.
**Lpin3**	4.15E-01	.	.	-1.01E+00	5.78E-01	.	.	3.51E-04
**Arhgdia**	6.86E-01	1.13E+00	.	.	3.93E-01	3.02E-17	.	.
**Samd14**	1.32E+00	.	.	-1.58E+00	6.06E-02	.	.	3.77E-07
**Gsg1l**	-3.43E-01	.	-1.02E+00	.	6.84E-01	.	6.42E-04	.
**Tmem100**	-2.10E-01	.	.	2.79E+00	7.45E-01	.	.	5.82E-13
**Gtf2a2**	-1.70E-02	.	-1.06E+00	.	9.84E-01	.	4.91E-03	.
**Ephb2**	8.00E-02	.	.	-1.06E+00	9.19E-01	.	.	2.05E-07
**Arl5c**	1.44E+00	.	.	-1.01E+00	1.00E-01	.	.	2.38E-07
**Gpr143**	-1.07E+00	.	.	2.62E+00	2.34E-01	.	.	6.17E-06
**Tmem88b**	2.57E-01	.	1.39E+00	.	4.57E-01	.	1.05E-08	.
**Serinc2**	-1.82E+00	.	.	2.68E+00	2.05E-01	.	.	4.44E-07
**Rbp1**	-4.88E-01	.	.	3.09E+00	2.81E-01	.	.	6.98E-16
**Calcb**	-1.68E-01	.	.	1.14E+00	7.73E-01	.	.	6.27E-16
**Edf1**	5.47E-01	1.28E+00	.	.	3.51E-01	4.40E-11	.	.
**Gcnt4**	-3.60E-02	.	.	3.46E+00	9.26E-01	.	.	3.18E-12
**Clec1b**	1.45E+00	.	.	-1.92E+00	2.02E-01	.	.	2.10E-03
**Klhl42**	4.10E-01	.	.	-1.39E+00	3.87E-01	.	.	5.60E-06
**Zscan22**	6.50E-02	1.20E+00	.	.	8.65E-01	3.82E-07	.	.
**Fabp3**	1.08E-01	.	.	-1.06E+00	9.39E-01	.	.	2.34E-10
**Dmtn**	3.78E-01	.	1.21E+00	.	2.89E-01	.	9.57E-08	.
**Mroh1**	8.96E-01	.	1.31E+00	.	2.66E-01	.	6.43E-04	.
**Tmem116**	-7.33E-01	.	.	2.91E+00	3.94E-01	.	.	5.69E-10
**Dnali1**	7.20E-02	.	1.31E+00	.	8.78E-01	.	2.22E-04	.
**Gm4013**	9.14E-01	.	1.35E+00	.	2.35E-01	.	3.81E-05	.
**Marveld3**	-5.93E-01	.	.	1.02E+00	2.83E-01	.	.	8.75E-04
**BC039966**	-8.21E-01	.	.	2.16E+00	4.13E-01	.	.	6.71E-37
**Tmem259**	9.95E-01	.	1.64E+00	.	1.91E-01	.	1.13E-05	.
**Strip2**	7.56E-01	.	1.70E+00	.	3.96E-01	.	7.18E-04	.
**Srek1ip1**	5.80E-02	1.75E+00	1.10E+00	.	8.88E-01	2.33E-23	5.58E-03	.
**Agbl2**	1.99E-01	.	.	-1.10E+00	8.60E-01	.	.	5.89E-04
**Aanat**	-1.40E+00	.	.	1.61E+00	1.26E-01	.	.	2.38E-06
**Tsr1**	1.50E-02	.	1.21E+00	.	9.76E-01	.	1.55E-03	.
**Ppfia4**	1.75E-01	.	.	-1.21E+00	7.09E-01	.	.	4.31E-05
**Chn1**	6.19E-01	.	3.19E+00	.	2.34E-01	.	7.85E-07	.
**Cdk8**	1.63E-01	.	.	-1.42E+00	7.52E-01	.	.	3.37E-04
**4921504E06Rik**	-5.32E-01	.	-1.13E+00	.	4.58E-01	.	2.63E-03	.

**Prickle2**	1.41E-01	.	2.60E+00	.	6.95E-01	.	1.44E-10	.
**Cmas**	-1.13E+00	.	.	2.16E+00	6.19E-01	.	.	5.54E-06
**Malat1**	4.58E-01	1.97E+00	.	.	1.87E-01	1.95E-24	.	.
**Ldoc1l**	1.48E-01	.	.	-1.38E+00	8.70E-01	.	.	7.45E-07
**Gpr133**	-3.47E-01	.	.	1.41E+00	5.09E-01	.	.	4.68E-22
**Rheb**	-1.97E-01	.	-1.12E+00	.	6.42E-01	.	7.79E-03	.
**Tmem138**	1.51E-01	1.16E+00	.	.	8.78E-01	2.48E-10	.	.
**Cenpl**	1.99E+00	1.30E+00	.	.	7.94E-02	8.93E-12	.	.
**Sec61a1**	4.08E-01	.	.	-1.11E+00	5.18E-01	.	.	8.73E-05
**A630075F10Rik**	-2.37E-01	.	.	2.66E+00	7.90E-01	.	.	1.70E-11
**Prkce**	1.05E+00	.	.	-1.85E+00	5.89E-02	.	.	4.14E-11
**Tbx3os2**	-1.07E+00	.	.	1.41E+00	2.46E-01	.	.	3.30E-07
**Sema3f**	1.51E+00	.	.	-1.01E+00	2.31E-01	.	.	4.98E-03
**Taldo1**	1.62E-01	.	1.24E+00	.	6.15E-01	.	4.68E-07	.
**Mcm2**	3.89E-01	.	1.88E+00	.	3.62E-01	.	2.70E-09	.
**Iffo2**	-8.76E-01	.	.	1.21E+00	2.52E-01	.	.	4.65E-06
**Cstf1**	7.31E-01	.	.	-1.15E+00	3.60E-01	.	.	2.12E-03
**Tppp3**	3.70E-01	.	.	-1.58E+00	6.55E-01	.	.	1.35E-04
**Zfp949**	9.45E-01	3.93E+00	2.18E+00	.	3.51E-01	9.91E-31	3.60E-18	.
**Slc4a1**	1.79E+00	.	.	-1.09E+00	2.15E-01	.	.	3.00E-06
**Cbfa2t3**	7.32E-01	.	.	-1.03E+00	3.07E-01	.	.	8.43E-09
**Scgb1c1**	-4.23E-01	.	.	1.01E+00	7.01E-01	.	.	1.18E-07
**Ckap2l**	1.45E+00	.	1.94E+00	.	9.47E-02	.	8.51E-06	.
**Zfp668**	1.01E+00	1.40E+00	.	.	1.68E-01	1.75E-11	.	.
**Fndc5**	1.42E-01	.	1.14E+00	.	6.46E-01	.	8.42E-05	.
**Lrp1**	4.21E-01	.	.	-1.38E+00	2.82E-01	.	.	3.28E-06
**Pfdn4**	-3.70E-01	.	.	3.28E+00	3.10E-01	.	.	1.40E-36
**Fgf3**	-2.84E-01	.	.	3.69E+00	6.31E-01	.	.	2.38E-21
**Cnn2**	1.67E+00	.	1.64E+00	.	6.17E-02	.	1.13E-05	.
**Rtfdc1**	-2.60E-01	.	.	1.35E+00	7.15E-01	.	.	4.25E-04
**Trnp1**	-3.63E-01	.	.	2.11E+00	6.14E-01	.	.	3.26E-12
**Gopc**	4.38E-01	1.33E+00	.	.	2.85E-01	1.18E-04	.	.
**Maneal**	1.09E+00	.	.	-1.13E+00	1.30E-01	.	.	2.14E-07
**Cdkn1c**	-2.54E-01	-1.17E+00	.	.	6.59E-01	3.28E-16	.	.
**Vash1**	-2.37E-01	.	-1.74E+00	.	5.70E-01	.	8.18E-05	.
**Cyth1**	1.21E+00	.	1.60E+00	.	6.30E-02	.	2.87E-06	.
**Shroom2**	-2.31E-01	.	.	5.21E+00	5.31E-01	.	.	1.69E-11
**Kcnj12**	-2.17E-01	.	-1.04E+00	.	6.81E-01	.	8.11E-04	.
**Fancl**	3.65E-01	1.13E+00	.	.	8.32E-01	2.51E-06	.	.
**Alg8**	1.84E+00	.	1.51E+00	.	4.38E-02	.	4.60E-04	.
**Mpdu1**	1.09E+00	.	1.98E+00	.	8.90E-02	.	6.68E-13	.

**Brinp2**	2.49E-01	.	.	-1.20E+00	8.31E-01	.	.	1.95E-04
**Tmtc2**	7.62E-01	.	.	-1.55E+00	1.82E-01	.	.	1.91E-03
**Ric3**	1.20E-01	.	.	-1.79E+00	7.49E-01	.	.	2.22E-04
**Cdc34**	3.37E-01	.	1.68E+00	.	7.09E-01	.	6.33E-13	.
**Slc30a10**	1.83E+00	.	.	-1.08E+00	5.26E-02	.	.	1.49E-09
**Zic1**	-3.48E-01	.	.	1.93E+00	5.75E-01	.	.	1.25E-18
**Slc39a9**	2.69E-01	.	1.65E+00	.	7.56E-01	.	5.78E-05	.
**Arl8a**	3.83E-01	.	.	-1.18E+00	3.32E-01	.	.	1.86E-03
**Mid1**	-9.32E-01	.	.	2.20E+00	4.85E-01	.	.	3.14E-09
**Tbc1d22a**	1.90E-02	.	1.57E+00	.	9.54E-01	.	9.91E-09	.
**Zfp608**	1.48E-01	.	1.58E+00	.	7.42E-01	.	5.84E-06	.
**Grpr**	-1.45E+00	.	.	3.74E+00	4.32E-02	.	.	5.30E-23
**Asic1**	9.04E-01	.	.	-1.44E+00	4.74E-02	.	.	8.05E-07
**Zbtb22**	1.49E-01	.	1.91E+00	.	7.28E-01	.	1.94E-06	.
**Timm10**	1.69E+00	.	3.03E+00	.	7.85E-02	.	1.14E-30	.
**Pid1**	1.53E-01	.	.	-1.23E+00	7.33E-01	.	.	2.11E-03
**Zfp940**	2.13E+00	.	1.74E+00	.	1.93E-01	.	1.25E-05	.
**Ccnd3**	7.60E-02	1.50E+00	.	.	9.30E-01	1.23E-21	.	.
**Pianp**	-2.44E-01	.	.	2.47E+00	6.32E-01	.	.	7.43E-06
**Cel**	9.95E-01	.	1.17E+00	.	1.91E-01	.	8.21E-07	.
**Cwc25**	5.27E-01	1.16E+00	.	.	5.10E-01	4.79E-13	.	.
**Cacng5**	-5.25E-01	.	.	3.83E+00	4.56E-01	.	.	3.02E-22
**2610306M01Rik**	6.60E-01	1.37E+00	.	.	3.04E-01	9.59E-15	.	.
**Itga3**	1.52E-01	.	.	-1.66E+00	8.75E-01	.	.	5.05E-06
**A130010J15Rik**	4.90E-02	.	.	-1.65E+00	9.01E-01	.	.	4.70E-04
**Bysl**	3.80E-02	.	1.62E+00	.	9.30E-01	.	5.61E-12	.
**4933439K11Rik**	-2.10E-01	.	.	1.52E+00	7.45E-01	.	.	1.95E-15
**Naa35**	4.90E-02	.	2.07E+00	.	9.10E-01	.	3.17E-14	.
**Cdk9**	3.84E-01	.	2.44E+00	.	3.28E-01	.	7.08E-13	.
**Pitpnm2**	1.28E-01	.	.	-1.08E+00	7.76E-01	.	.	3.43E-06
**Marcks**	-8.00E-03	.	-1.34E+00	.	9.85E-01	.	1.48E-05	.
**Gm1564**	6.66E-01	-1.38E+00	.	-1.30E+00	5.60E-01	1.75E-10	.	5.52E-03
**Sfn**	1.46E-01	.	.	-1.08E+00	8.76E-01	.	.	2.87E-06
**Eci3**	8.02E-01	1.33E+00	1.44E+00	.	4.05E-01	1.33E-08	1.08E-05	.
**Dusp15**	4.68E-01	.	.	-1.21E+00	7.08E-01	.	.	3.72E-06
**Myadm**	1.09E+00	.	1.20E+00	.	3.81E-02	.	5.36E-03	.
**Paqr9**	2.80E-01	.	.	-1.11E+00	5.18E-01	.	.	1.38E-11
**3200001D21Rik**	1.60E+00	.	1.59E+00	.	1.14E-01	.	3.79E-07	.
**Suclg2**	5.10E-02	.	.	-1.79E+00	8.80E-01	.	.	8.18E-05
**Alkbh6**	9.60E-02	.	2.14E+00	.	8.05E-01	.	1.30E-08	.

**Tmprss12**	5.65E-01	.	.	-1.45E+00	3.61E-01	.	.	6.86E-04
**Slc25a17**	7.20E-02	1.09E+00	.	.	8.45E-01	1.29E-09	.	.
**Oser1**	1.54E-01	1.01E+00	.	.	7.88E-01	2.29E-06	.	.
**Calca**	-1.40E-01	.	.	1.17E+00	6.98E-01	.	.	2.34E-11
**Ipmk**	2.74E-01	.	1.05E+00	.	5.20E-01	.	5.19E-05	.
**Tbrg4**	1.74E-01	.	1.19E+00	.	8.74E-01	.	2.85E-06	.
**9530080O11Rik**	5.38E-01	.	.	-1.25E+00	4.46E-01	.	.	2.09E-06
**Mrgpre**	-1.01E-01	.	.	1.90E+00	9.15E-01	.	.	1.78E-06
**Tcp1**	9.20E-01	1.02E+00	.	.	3.89E-01	1.67E-10	.	.
**Ddx17**	1.07E+00	.	1.22E+00	.	1.65E-01	.	9.40E-03	.
**Bhlha15**	8.87E-01	.	.	-1.30E+00	2.72E-01	.	.	6.48E-08
**Phb**	-2.25E-01	.	.	2.52E+00	5.24E-01	.	.	5.96E-10
**S100a1**	5.60E-02	1.22E+00	.	.	9.34E-01	8.69E-13	.	.
**Lrrc4c**	2.19E-01	.	.	-1.37E+00	7.48E-01	.	.	2.45E-04
**Scn2b**	1.21E-01	1.20E+00	.	.	7.92E-01	2.99E-09	.	.
**Lepr**	1.79E-01	.	.	-1.04E+00	6.46E-01	.	.	1.07E-03
**Gpx7**	4.41E-01	.	.	-1.22E+00	7.10E-01	.	.	1.12E-03
**Ctxn2**	-4.36E-01	.	.	2.21E+00	2.38E-01	.	.	3.75E-08
**Fscn1**	-1.21E-01	.	.	1.17E+00	7.26E-01	.	.	1.10E-04
**Evi5l**	7.50E-02	.	1.80E+00	.	9.24E-01	.	1.41E-05	.
**Tmem87a**	5.14E-01	2.34E+00	.	.	1.80E-01	5.43E-19	.	.
**Akt2**	1.22E+00	.	1.17E+00	.	6.28E-02	.	6.69E-06	.
**Cdca3**	3.80E-01	.	2.72E+00	.	7.88E-01	.	2.37E-09	.
**Mon2**	-3.37E-01	.	-1.25E+00	.	3.42E-01	.	2.67E-03	.
**Irf9**	6.84E-01	.	1.49E+00	.	1.56E-01	.	2.96E-06	.
**Slc38a2**	2.53E-01	1.02E+00	.	.	5.67E-01	2.17E-09	.	.
**Dclre1c**	1.71E+00	2.03E+00	.	.	1.89E-01	3.31E-17	.	.
**Tfb2m**	2.86E-01	.	1.97E+00	.	5.41E-01	.	6.95E-09	.
**Esrra**	2.55E-01	.	1.27E+00	.	4.24E-01	.	3.52E-06	.
**Cdh26**	-2.09E+00	.	.	1.30E+00	4.40E-02	.	.	1.35E-08
**Lpin2**	1.09E-01	1.00E+00	.	.	7.91E-01	6.41E-04	.	.
**Ormdl2**	-8.85E-01	.	-1.25E+00	.	3.33E-01	.	2.70E-03	.
**Ptpre**	4.22E-01	.	.	-1.13E+00	2.71E-01	.	.	1.45E-04
**Ccrl2**	7.86E-01	1.13E+00	.	.	4.28E-01	1.27E-10	.	.
**Thbs2**	1.62E-01	.	1.11E+00	.	7.32E-01	.	4.90E-04	.
**Rimkla**	2.35E-01	.	.	-1.78E+00	6.65E-01	.	.	6.47E-06
**Nr1h3**	2.37E-01	.	.	-1.18E+00	6.62E-01	.	.	1.91E-04
**Grik3**	5.59E-01	.	1.06E+00	.	3.42E-01	.	1.52E-03	.
**D430041D05Rik**	5.38E-01	.	.	-1.27E+00	1.84E-01	.	.	1.12E-07
**Ddb2**	2.24E+00	.	2.40E+00	.	7.65E-02	.	3.31E-06	.
**Rgs19**	2.20E-01	.	.	-1.05E+00	7.94E-01	.	.	3.12E-06

**Serpinh1**	7.71E-01	.	1.02E+00	.	3.86E-01	.	1.87E-05	.
**Mmp24**	6.24E-01	.	.	-1.05E+00	3.22E-01	.	.	4.48E-07
**Eme1**	4.20E-02	1.35E+00	.	.	9.59E-01	1.74E-07	.	.
**Vac14**	1.66E-01	.	1.88E+00	.	7.48E-01	.	1.15E-12	.
**Clic4**	-9.00E-02	.	1.47E+00	1.06E+00	7.90E-01	.	5.98E-09	2.45E-03
**Spen**	1.37E-01	.	1.08E+00	.	7.73E-01	.	9.04E-06	.
**Sp7**	2.03E+00	.	1.11E+00	.	4.18E-02	.	1.98E-04	.
**Cxxc4**	8.30E-02	.	.	-2.82E+00	8.37E-01	.	.	7.43E-06
**Ankrd34a**	9.28E-01	.	.	-1.29E+00	1.58E-01	.	.	3.45E-08
**Slc12a5**	4.10E-02	.	.	-1.07E+00	9.20E-01	.	.	5.63E-04
**Tet2**	3.30E-02	1.92E+00	1.25E+00	.	9.51E-01	6.17E-14	2.13E-08	.
**Prmt8**	-1.11E+00	.	.	2.54E+00	2.52E-01	.	.	2.58E-07
**Nalcn**	6.09E-01	.	.	-1.43E+00	2.46E-01	.	.	1.18E-05
**Crip1**	9.53E-01	.	.	-1.22E+00	4.84E-01	.	.	1.56E-03
**Hunk**	-4.40E-02	.	.	3.56E+00	9.39E-01	.	.	1.32E-17
**Ptbp1**	6.59E-01	.	1.39E+00	.	4.61E-01	.	9.33E-09	.
**Fbxl5**	-4.34E-01	-1.05E+00	.	.	4.43E-01	1.61E-03	.	.
**Adrbk2**	1.23E+00	.	.	-1.04E+00	8.23E-02	.	.	5.22E-04
**Zc3hc1**	3.00E-02	1.91E+00	.	.	9.59E-01	8.85E-14	.	.
**Myo9a**	1.30E-02	.	1.69E+00	.	9.74E-01	.	4.77E-07	.
**Raver2**	-4.93E-01	.	.	1.80E+00	5.84E-01	.	.	2.72E-04
**Asprv1**	2.66E-01	.	.	-1.37E+00	8.63E-01	.	.	1.42E-04
**Ak4**	4.48E-01	.	.	-1.80E+00	3.28E-01	.	.	1.34E-08
**Wdr81**	2.11E+00	.	2.49E+00	.	2.35E-02	.	5.05E-09	.
**Fignl2**	-1.03E-01	.	.	1.15E+00	8.09E-01	.	.	3.20E-11
**Btbd2**	2.37E-01	.	1.41E+00	.	6.49E-01	.	1.04E-06	.
**Mrpl27**	8.96E-01	1.35E+00	.	.	3.08E-01	1.74E-07	.	.
**Fastkd5**	6.73E-01	.	.	-1.28E+00	4.32E-01	.	.	1.41E-21
**Nyap1**	1.35E+00	.	.	-1.28E+00	8.68E-02	.	.	9.28E-05
**Wscd2**	4.23E-01	.	.	-1.09E+00	4.21E-01	.	.	1.21E-04
**Kifc3**	6.67E-01	.	.	-1.29E+00	4.09E-01	.	.	6.61E-03
**Adap1**	3.75E-01	.	.	-1.94E+00	6.04E-01	.	.	2.49E-03
**Gm166**	6.86E-01	.	1.12E+00	.	3.82E-01	.	1.16E-04	.
**C330006A16Rik**	1.70E-01	.	1.49E+00	.	8.12E-01	.	2.77E-05	.
**Ahdc1**	8.90E-02	.	.	-1.37E+00	8.57E-01	.	.	2.17E-04
**Stambp**	7.70E-02	.	1.51E+00	.	8.32E-01	.	4.70E-07	.
**Dner**	3.97E-01	.	.	-1.18E+00	4.26E-01	.	.	8.45E-09
**Mrpl32**	8.29E-01	2.04E+00	.	.	4.38E-01	3.81E-09	.	.
**Alkbh4**	4.30E-01	.	1.22E+00	.	4.17E-01	.	1.86E-03	.
**Med18**	8.60E-01	1.20E+00	.	.	5.68E-01	7.26E-10	.	.
**Ppif**	8.10E-02	.	1.58E+00	.	8.43E-01	.	2.49E-06	.
**Rbm12b2**	1.75E-01	1.82E+00	.	.	6.63E-01	3.05E-15	.	.

**Foxp1**	3.42E-01	.	1.67E+00	.	4.83E-01	.	1.30E-05	.
**Cntd1**	1.47E-01	.	.	-1.17E+00	7.91E-01	.	.	3.18E-05
**Robo3**	1.02E-01	.	1.21E+00	.	9.02E-01	.	2.77E-03	.
**Trim67**	2.81E-01	.	.	-1.12E+00	5.23E-01	.	.	5.07E-19
**Gm16701**	1.42E+00	.	.	-1.25E+00	1.02E-01	.	.	6.99E-06
**Rnf152**	2.73E-01	.	.	-1.27E+00	4.68E-01	.	.	1.88E-07
**Fam83d**	1.32E+00	.	1.13E+00	.	1.37E-01	.	1.03E-05	.
**Il4ra**	-8.52E-01	.	.	1.30E+00	3.36E-01	.	.	9.20E-10
**6530411M01Rik**	1.79E-01	.	2.26E+00	.	8.17E-01	.	6.23E-08	.
**Bcl6b**	1.52E-01	1.00E+00	.	.	8.40E-01	1.06E-08	.	.
**Zfp58**	4.58E-01	1.45E+00	.	.	6.77E-01	1.34E-16	.	.
**Ddx10**	4.31E-01	.	1.62E+00	.	4.98E-01	.	1.02E-04	.
**Sp2**	2.63E+00	.	.	-1.42E+00	1.37E-02	.	.	5.58E-05
**Lrp8**	3.49E-01	.	.	-1.14E+00	4.48E-01	.	.	1.45E-06
**Gltp**	7.18E-01	2.12E+00	.	.	3.94E-01	1.42E-29	.	.
**Zfp286**	6.70E-02	1.18E+00	.	.	9.20E-01	2.55E-12	.	.
**Sarnp**	-2.61E-01	.	-1.25E+00	.	4.47E-01	.	2.70E-03	.
**Star**	1.20E+00	.	1.51E+00	.	1.07E-01	.	1.86E-06	.
**Phf21a**	1.45E+00	1.07E+00	.	.	6.38E-02	1.13E-05	.	.
**Scara5**	1.02E+00	.	1.39E+00	.	2.21E-01	.	2.34E-04	.
**Ncapg2**	4.75E-01	.	1.05E+00	.	4.24E-01	.	2.04E-04	.
**Prex1**	-3.38E-01	.	.	1.44E+00	5.16E-01	.	.	6.38E-08
**Ppfibp1**	6.04E-01	.	.	-1.13E+00	5.54E-01	.	.	7.02E-03
**Pde1b**	2.78E-01	.	.	-1.10E+00	8.36E-01	.	.	4.44E-09

Table S5: Gene Ontology enrichments for 145 genes in the active circuit after network analysis.

GO terms are scored by EnrichR combined score.

Category	Term	Overlap	Combined Score	Genes
**GO Biological Process**	regulation of dendritic spine morphogenesis (GO:0061001)	4/21	1.133703336	EFNA1 SRCIN1 SHANK3 LRP8
	negative regulation of cellular component organization (GO:0051129)	21/474	1.097462581	RTN4R PSMD10 PID1 DMTN ITGA3 SHROOM2 NR1H3 SEMA3F MID1 CNN2 EFNA1 RPS6KA2 TMSB4X ARHGDIA MYADM PPIF PACSIN3 GOPC EVI5L EPHB2 SHANK3
	actin cytoskeleton organization (GO:0030036)	14/253	1.02254391	CAP1 MON2 DMTN IQSEC2 IQSEC1 ARHGAP26 CNN3 FOXP1 CNN2 ALKBH4 NUAK2 DAAM2 TMSB4X FSCN1
	calcium-mediated signaling using intracellular calcium source (GO:0035584)	3/13	1.020641134	FIS1 GPR143 DMTN
	cell-cell junction maintenance (GO:0045217)	3/9	1.007143753	SHROOM2 MYADM KIFC3
	terpenoid metabolic process (GO:0006721)	8/97	0.992727145	AKR1B10 LRP1 STAR AWAT2 RBP1 PDE3A LRP8 GPC6
	diterpenoid metabolic process (GO:0016101)	8/87	0.986640404	AKR1B10 LRP1 STAR AWAT2 RBP1 PDE3A LRP8 GPC6
	cellular response to metal ion (GO:0071248)	8/99	0.979017033	CLIC4 DMTN STAR SLC11A2 PPIF PPARG AQP2 JUNB
	cellular response to antibiotic (GO:0071236)	3/13	0.970089779	XBP1 STAR CRIP1
	cell junction maintenance (GO:0034331)	3/11	0.938976983	SHROOM2 MYADM KIFC3
	regulation of cell morphogenesis involved in differentiation (GO:0010769)	13/248	0.892376823	RTN4R DMTN SEMA3F LRP8 SRCIN1 EFNA1 PREX1 ARHGDIA CHN1 MYADM LRRC4C EPHB2 SHANK3
**GO Molecular Function**	ARF guanyl-nucleotide exchange factor activity (GO:0005086)	4/19	4.979041721	MON2 IQSEC2 IQSEC1 CYTH1
	actin binding (GO:0003779)	19/386	4.623746741	CAP1 DMTN GMFG PRKCE SHROOM2 SLC4A1 AQP2 MYO9A CNN3 CNN2 MARCKS ALKBH4 DAAM2 HOOK1 TMSB4X FSCN1 SHANK3 MSRB1 PLS1
	sodium channel activity (GO:0005272)	5/37	4.281021029	SCNN1B SHROOM2 NALCN SCN2B ASIC1
	ligand-gated sodium channel activity (GO:0015280)	3/10	3.904128246	SCNN1B SHROOM2 ASIC1
	ligand-activated sequence-specific DNA binding RNA polymerase II transcription factor activity (GO:0004879)	5/50	2.509575238	ESRRA NR5A2 NR1H3 PPARG BRD8
	direct ligand regulated sequence-specific DNA binding transcription factor activity (GO:0098531)	5/50	2.49141456	ESRRA NR5A2 NR1H3 PPARG BRD8
	mRNA 3'-UTR binding (GO:0003730)	4/35	1.82862356	CPEB1 ELAVL4 RNPS1 ELAVL2
	RNA helicase activity (GO:0003724)	3/29	1.447110189	DDX17 DHX8 DDX10

	GTPase regulator activity (GO:0030695)	13/310	1.397224764	GPSM1 RGS19 ARHGAP26 ADAP1 MYO9A RASGRP3 PREX1 ARHGDIA CHN1 GOPC CHML EVI5L TBC1D22A
	nucleoside-triphosphatase regulator activity (GO:0060589)	13/330	1.385629816	GPSM1 RGS19 ARHGAP26 ADAP1 MYO9A RASGRP3 PREX1 ARHGDIA CHN1 GOPC CHML EVI5L TBC1D22A
	small GTPase regulator activity (GO:0005083)	9/195	1.358239818	GPSM1 PREX1 ARHGDIA GOPC EVI5L ARHGAP26 ADAP1 TBC1D22A RASGRP3
**GO Cellular Component**	cortical actin cytoskeleton (GO:0030864)	3/24	0.900972322	CAP1 SHROOM2 MYADM
	synapse part (GO:0044456)	15/395	0.829575204	CALCA CLSTN3 GRIK3 FAIM2 LRP8 SRCIN1 CPEB1 PPFIBP1 GOPC LRRC4C PPFIA4 GABRD SHANK3 CBLN1 CACNG5
	postsynaptic membrane (GO:0045211)	11/195	0.826774805	CPEB1 CLSTN3 GRIK3 GOPC FAIM2 LRRC4C GABRD SRCIN1 SHANK3 CBLN1 CACNG5
	synaptic membrane (GO:0097060)	11/228	0.820510025	CPEB1 CLSTN3 GRIK3 GOPC FAIM2 LRRC4C GABRD SRCIN1 SHANK3 CBLN1 CACNG5
	cortical cytoskeleton (GO:0030863)	5/50	0.810816311	CAP1 DMTN MYADM SHROOM2 SLC4A1
	nucleoplasm (GO:0005654)	30/1051	0.739154106	PSMD10 GTF2A2 NCAPG2 PHB PHF21A PTBP1 RPS6KA2 CSTF1 BRD8 JUNB ESRRA CENPU XBP1 FANCL IPMK NR1H3 VRK1 SMAD5 DDB2 CDK9 RAD51B CDK8 NR5A2 MAPKAPK3 MED20 RNPS1 PPARG KIF20A IRF9 MCM2
	occluding junction (GO:0070160)	6/108	0.736582186	CLDN15 MAGI3 MARVELD3 SHROOM2 CLDN2 CYTH1
	apical part of cell (GO:0045177)	5/87	0.729870135	CLIC4 FZD7 SLC11A2 VASH1 BYSL
	tight junction (GO:0005923)	6/108	0.729224892	CLDN15 MAGI3 MARVELD3 SHROOM2 CLDN2 CYTH1
	neuronal cell body (GO:0043025)	10/270	0.711029059	CALCA SLC12A5 STAR LRP1 RHEB PDE1B DNER SHROOM2 EPHB2 LRP8
	clathrin-coated vesicle (GO:0030136)	4/57	0.699084763	RGS19 LRP1 GOPC AQP2

Table S6: Summary statistics of SAGE sequencing. For each sample we report the number of mapped tags, the number of total sequencing tags and the percentage of aligned tags

Samples	Mapped Tags	Total Sequences	Mapping %
**1CT**	10,204,508	53,290,259	19.15
**2CT**	4,170,458	31,517,774	13.23
**3CT**	7,035,817	50,823,964	13.84
**4CT**	4,446,023	32,505,049	13.68
**5CT**	5,753,513	34,003,519	16.92
**1RCF**	1,379,561	31,147,342	4.43
**2RCF**	6,945,345	40,705,489	17.06
**3RCF**	1,581,025	35,345,749	4.47
**4RCF**	5,168,060	37,911,879	13.63
**5RCF**	5,218,794	45,043,440	11.59
**Total**	**51,903,104**	**392,294,464**	**13.23**
